# Spatiotemporally Controlled T‐Cell Combination Therapy for Solid Tumor

**DOI:** 10.1002/advs.202401100

**Published:** 2024-04-17

**Authors:** Meixi Hao, Ying Zhou, Sijia Chen, Yu Jin, Xiuqi Li, Lingjing Xue, Mingxuan Shen, Weishuo Li, Can Zhang

**Affiliations:** ^1^ State Key Laboratory of Natural Medicines Center of Advanced Pharmaceuticals and Biomaterials China Pharmaceutical University Nanjing 211198 China; ^2^ Chongqing Innovation Institute of China Pharmaceutical University Chongqing 401135 China; ^3^ Center for Molecular Metabolism School of Environmental and Biological Engineering Nanjing University of Science and Technology 200 Xiao Ling Wei Street Nanjing 210094 China

**Keywords:** core‐shell structured nanoparticle, site‐specific drug release, spatiotemporally controlled cytopharmaceutical, T‐cell therapy, triple drug combination

## Abstract

Due to multidimensional complexity of solid tumor, development of rational T‐cell combinations and corresponding formulations is still challenging. Herein, a triple combination of T cells are developed with Indoleamine 2,3‐dioxygenase inhibitors (IDOi) and Cyclin‐dependent kinase 4/6 inhibitors (CDK4/6i). To maximize synergism, a spatiotemporally controlled T‐cell engineering technology to formulate triple drugs into one cell therapeutic, is established. Specifically, a sequentially responsive core‐shell nanoparticle (SRN) encapsulating IDOi and CDK4/6i is anchored onto T cells. The yielded SRN‐T cells migrated into solid tumor, and achieved a 1st release of IDOi in acidic tumor microenvironment (TME). Released IDOi restored tryptophan supply in TME, which activated effector T cells and inhibited Tregs. Meanwhile, 1st released core is internalized by tumor cells and degraded by glutathione (GSH), to realize a 2nd release of CDK4/6i, which induced up‐regulated expression of C‐X‐C motif chemokine ligand 10 (CXCL10) and C‐C motif chemokine ligand 5 (CCL5), and thus significantly increased tumor infiltration of T cells. Together, with an enhanced recruitment and activation, T cells significantly suppressed tumor growth, and prolonged survival of tumor‐bearing mice. This study demonstrated rationality and superiority of a tri‐drug combination mediated by spatiotemporally controlled cell‐engineering technology, which provides a new treatment regimen for solid tumor.

## Graphical abstract

Spatiotemporally controlled SRN‐T cells achieved a 1st release of Indoleamine 2,3‐dioxygenase inhibitors (IDOi) in an acidic tumor microenvironment (TME). The released IDOi restored tryptophan supply in TME, which activated effector T cells and inhibited Tregs. Meanwhile, 1st released core is internalized by tumor cells and degraded by glutathione (GSH), to realize a 2nd release of Cyclin‐dependent kinase 4/6 inhibitors (CDK4/6i), which induces up‐regulated expression of C‐X‐C motif chemokine ligand 10 (CXCL10) and C‐C motif chemokine ligand 5 (CCL5), and thus a significantly increased tumor infiltration of T cells. Together, with an enhanced recruitment and activation, T cells significantly suppress tumor growth.

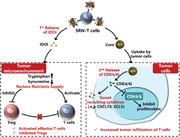

## 1 Introduction

Adoptive T‐cell therapy (ACT) represents an unprecedented breakthrough in the field of tumor treatment.^[^ ^1^, ^2^, ^3^, ^4^ ^]^ In particular, T‐cells engineered to express Chimeric Antigen Receptors (CARs) have demonstrated significant clinical efficacy for treating hematological malignancies.^[^ ^5^, ^6^ ^]^ However, the success of Chimeric Antigen Receptor T cell (CAR‐T) in solid tumors has been challenged by the tumor‐antigen heterogeneity, impaired CAR‐T cell trafficking and infiltration, and presence of an immunosuppressive tumor microenvironment (TME), etc.^[^ ^5^, ^7^, ^8^ ^]^ Combining T cells with other therapeutic modalities might hold great promise in improving the effectiveness of CAR‐T against solid tumors.^[^ ^4^, ^8^, ^9^, ^10^ ^]^ Currently, several T‐cell combination therapies are undergoing clinical trials.^[^ ^5^ ^]^ Notably, most of these combinations are focusing on two‐drug combinations, including but not limited to chemotherapeutic with T cells, and immune checkpoint inhibitors with T cells, etc.^[^ ^5^ ^]^ However, multidimensional complexity of solid tumor, particularly the TME, necessities the development of multiple drug combination to better break the tumor immunosuppression and thus conquer solid tumor to the best extent.^[^ ^11^ ^]^

Indoleamine 2,3‐dioxygenase (IDO) in the TME produced by tumor cells and immunosuppressive cells, is an immunoregulatory enzyme that decomposes tryptophan (Trp) into metabolic byproducts known as kynurenines (Kyn). The depletion of Trp and accumulation of Kyn in the TME results in suppression of effector T cells, meanwhile promoting the function of T regulatory. Therefore, IDO inhibitors (IDOi) can overcome the immunosuppressive TME and promote activation of T cells.^[^ ^12^ ^]^ Notably, the combination of the IDOi and CAR‐T cells showed improved tumor control over lymphoma,^[^ ^13^ ^]^ which suggested that IDOi can promote activation of CAR‐T cells. However, no clinical benefits of IDOi and CAR‐T cells combination were observed in solid tumors.^[^ ^14^ ^]^ That might because solid tumors, especially those “cold” T cell‐excluded tumors, e.g. melanoma, often have low levels of tumor infiltrated T cells,^[^ ^15^ ^]^ partly contributing to this failure of clinical trials of IDOi and CAR‐T cells combination therapy.

Trafficking and localization of T cells is mainly regulated by C‐X‐C motif chemokine receptor 3 (CXCR3)^[^ ^16^ ^]^ following gradients of C‐X‐C motif chemokine ligand (CXCL) 9,^[^ ^17^ ^]^ 10,^[^ ^18^ ^]^ or 11.^[^ ^19^, ^20^ ^]^ These chemokines are secreted by tumor cells and non‐malignant cells within the TME, determining the numbers of tumor‐infiltrating T cells.^[^ ^21^ ^]^ However, the expression of CXCL9, 10, or 11 in most tumors is limited, which has been linked with poor T cells infiltration.^[^ ^18^ ^]^ Cyclin‐dependent kinase 4 and 6 (CDK4/6) are critical mediators of cellular transition into S phase of the cell cycle. Aberrant activity of CDK4/6 has been demonstrated to lead to uncontrolled cell proliferation, which have been observed in a variety of tumors. It has been demonstrated that pharmacological inhibitors of CDK4/6, could directly arresting tumor cells in the G1 phase of the cell cycle.^[^ ^22^ ^]^ Additionally, CDK4/6 inhibitors (CDK4/6i) could modulate anti‐tumor immunity in several ways. For example, CDK4/6i promotes recruitment of T cells into solid tumors by inducing expression of T‐cell chemotactic chemokines, e.g. CXCL10 and C‐C motif chemokine ligand 5 (CCL5), in tumor cells.^[^ ^23^ ^]^ Furthermore, treating tumor cells with CDK4/6i facilitates their recognition by the immune system via enhancing expression of major histocompatibility complex class I (MHC‐I).^[^ ^24^, ^25^ ^]^

**Figure 1:**
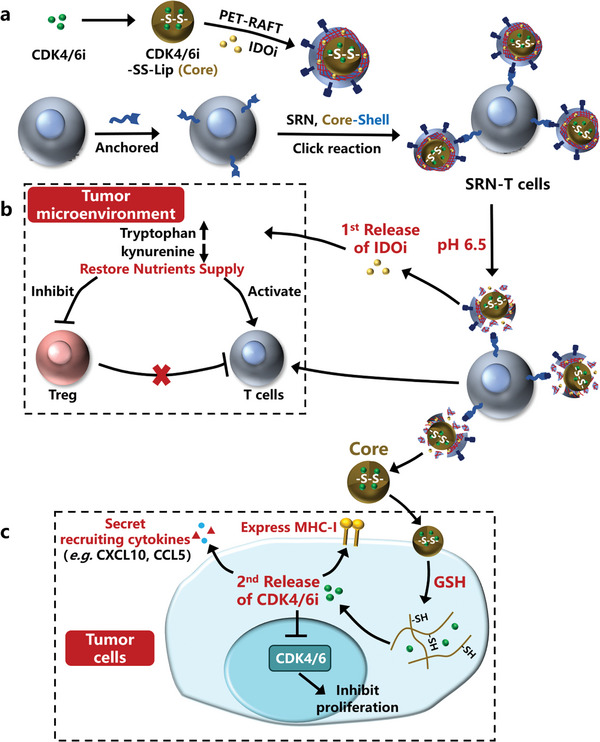
Schematic diagram of spatiotemporally controlled T‐cell combination therapy. a) Fabrication of SRN‐T cells. b) 1st release of IDOi from pH‐ruptured shell in TME. c) 2nd release of CDK4/6i in tumor cells with a GSH‐responsive manner.

Therefore, we hypothesized that combining CDK4/6i with T cell and IDOi would solve the issue (poor infiltration of T cells) in the scenario of IDOi‐T cell combination therapy. Meanwhile, this proposed triple drug combination would achieve an enhanced therapeutic outcome toward solid tumors. To maximize synergism among this triple‐drug combination, we established a novel spatiotemporally controlled T‐cell engineering technology, based on which a cell therapeutic of T cells, IDOi and CDK4/6i, were fabricated. Specifically, core‐shell structured nanoparticles (SRN) were first fabricated with IDOi in pH‐sensitive shell and CDK4/6i in Glutathione (GSH)‐sensitive core. Then SRN were anchored onto T cells to yield the SRN‐T cells (**Figure** **1**a). After an intravenous injection, SRN‐T cells migrated into solid tumor, and achieved a 1st drug release in the TME due to the acidic pH. The 1st released IDOi, restored Trp supply in TME, which activated effector T cells, and inhibited Tregs (Figure 1b). At the same time, the 1st released core was internalized by tumor cells and degraded by intracellular GSH, to realize a 2nd release of CDK4/6i in tumor cells, which induced an up‐regulated expression of CXCL10 and CCL5, and thus a significantly increased tumor infiltration of T cells (Figure 1c). Together, with an enhanced recruitment and activation, 1st released T cells significantly suppressed tumor growth, and prolonged the survival of tumor‐bearing mice. Furthermore, the endogenous anti‐tumor immunity was also activated. Our study demonstrated the great therapeutic potential of a triple‐drug combination for solid tumor immunotherapy, on the basis of a novel spatiotemporally controlled cell‐engineering technology.

## 2 Results and Discussion

### 2.1 Fabrication and Characterization of SRN‐T cells

**Figure 2:**
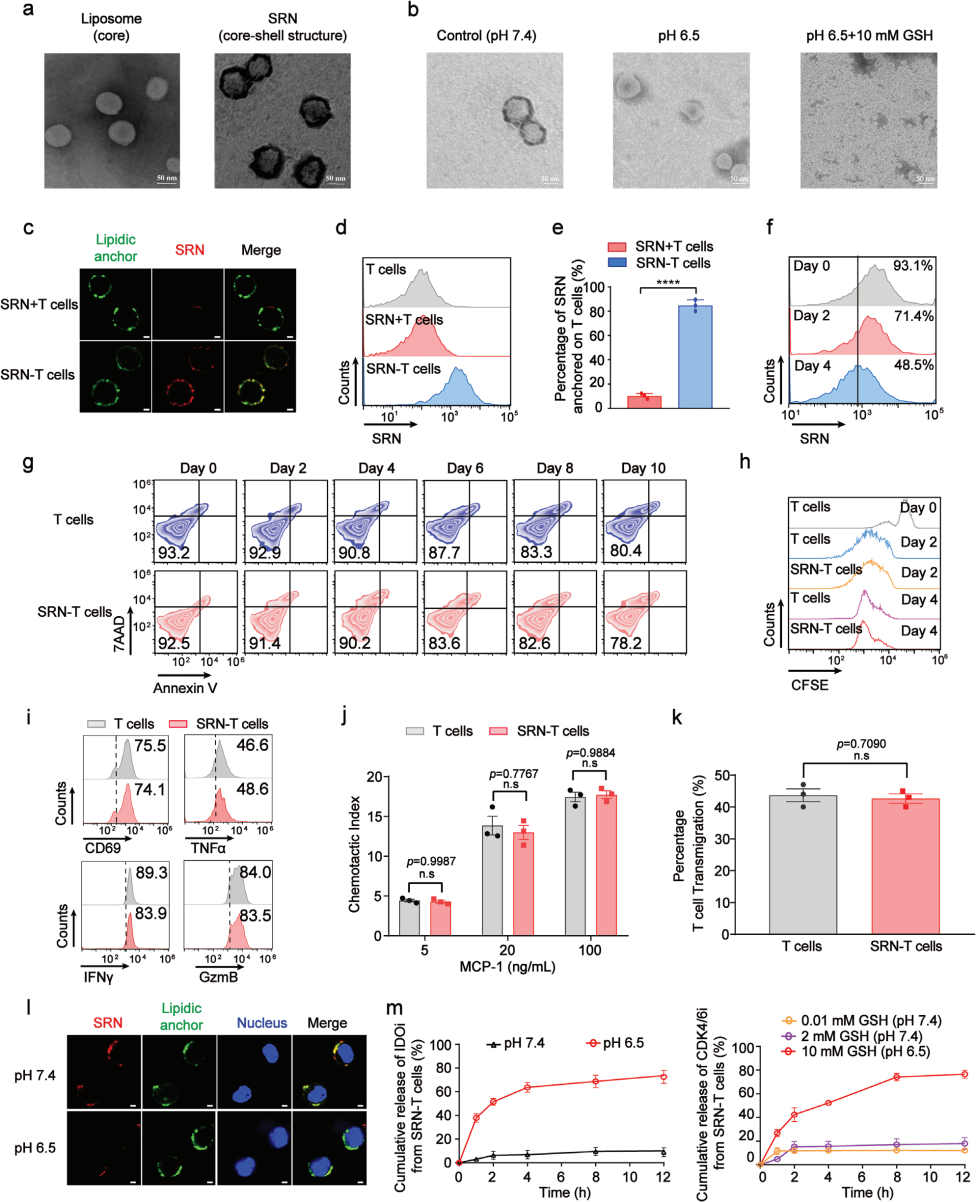
Formulating triple drugs into spatiotemporally controlled SRN‐T cells. a) TEM images of liposome and SRN. Scale bar: 50 nm. b) TEM images of SRN when incubated with different stimuli. Scale bar: 50 nm. c) Confocal microscopy images of SRN‐T cells. Rhodamine B labeled SRN (SRN‐RhoB, red), the clickable lipidic anchor (green), T cells (blue). Scale bar: 2 µm. d) Flow cytometry analysis of SRN‐T cells. SRN‐RhoB (red). e) The anchorage efficiency of SRN onto T cells. f) Flow cytometry analysis of SRN‐T cells. g) Representative flow cytometric analysis of the cell viability of SRN‐T cells during in vitro expansion. h) The proliferation capacity of SRN‐T cells stimulated with anti‐CD3/C28 beads, using carboxyfluorescein succinimidyl ester (CFSE) as indicator. i) Representative flow cytometric analysis of expression of CD69, TNFα, IFNγ, and GzmB in SRN‐T cells after stimulation. Chemotactic j) and transvascular migration k) of SRN‐T cells. l) Confocal microscopy images of SRN‐T cells under different pHs. m) In vitro spatiotemporally controlled drug release behavior of SRN‐T cells. Data are represented as mean ± SEM; n = 3; ^****^ *p* < 0.0001; n.s, not significant; determined by unpaired *t* test in e, j, k.

Two drugs, IDOi and CDK4/6i, were first formulated into SRN, consisting of a GSH‐degradable liposomal core and an acid‐cleavage polymeric shell. To do so, a lipidic chain transfer agent (CTA) with responsiveness to GSH was synthesized (Figure S1a, Supporting Information), of which chemical structure was verified by proton nuclear magnetic resonance (^1^H‐NMR) and high‐resolution mass spectrometry (HRMS, Figure S1b, Supporting Information). Then core of SRN, GSH‐responsive liposomal CDK4/6i (abemaciclib), was made from synthesized lipidic CTA, displaying a size of ≈86.9 nm and CTA on the surface (Figure S2a, CDK4/6i‐loading rate: ≈2%). Next reversible addition‐fragmentation chain transfer polymerization (PET‐RAFT)^[^ ^26^ ^]^ was conducted on the surface of liposomal core, using acrylamide (AAm) and clickable PEG (DBCO functionalized PEG) as monomers, acid‐cleavage glycerol dimethacrylate as the cross‐linker. After a 30‐min polymerization, the size of the yielded product was measured at ≈143.6 nm, demonstrating a clickable polymeric shell was formed on the surface of liposomal core (Figure S2a,b, Supporting Information). By simply dispersing IDOi into the PET‐RAFT system, nearly 89.4% of fielded IDOi was entrapped into the polymeric shell with a loading rate of ∼1.95%. To further verify the core‐shell structure of SRN, morphology of SRN was studied by transmission electron microscopy (TEM). As displayed in **Figure** **2**a, SRN were consisted of a 90‐nm liposomal core and a uniform polymeric shell with a thickness of 50 nm. The sequential responsiveness of SRN was then investigated by TEM. As shown in Figure 2b, the polymeric shell of SRN was degraded under acidic environment (pH 6.5). The yielded 50 nm nano core was degraded into fragments when further incubated with GSH (10 mM). We then investigated stability of SRN under physiology‐related conditions, especially with existence of abundant proteins. As displayed in Figure S3a (Supporting Information), the size of SRN dispersed in 1640 completed medium remained almost unchanged. Moreover, no significant particle size change and drug leak was observed at different temperature up to 72 h (Figure S3b–f, Supporting Information).

To anchor the SRN onto T‐cell surface, we first inserted a clickable lipidic anchor onto T‐cell surface based on our previous study.^[^ ^27^ ^]^ The concentration and insertion time of lipidic anchor (DPPE‐PEG_5k_‐N_3_) was fixed at 20 µm and 10 min, respectively, at which the lipidic anchor did not cause a significant loss of T cell viability (Figure S4a–d, Supporting Information). Then SRN (CDK4/6i concentration: 100 µg mL^−1^, at which concentration T cells remained viable, Figure S4e–h, Supporting Information) were incubated with T cells‐displaying lipidic anchor for 45 min. We then verified the successful anchorage of SRN by confocal microscopy. As displayed in Figure 2c, the lipidic anchor tagged by FITC was inserted evenly onto cell surface, and SRNs labeled by Rhodamine B (SRN‐RhoB) were subsequently clicked onto cell surface, which co‐localized well with the lipidic anchor. Notably, when using the lipidic anchor lack of clickable group as control, there were not much SRN‐RhoB observed on cell surface, demonstrating the click chemistry instead of physical interaction took a key role during the anchorage. Similarly, the results of flow cytometry were in consistent with confocal microscope, most of the SRN‐RhoB anchoring onto cell surface through the lipidic anchor with clickable group, rather than those without clickable group (Figure 2d). Further, we quantified the anchorage of SRN onto cell surface by nanoparticle tracking analysis. Approximately 226 SRNs were averagely anchored onto every T‐cell surface, namely ≈4.8 µg CDK4/6i and 4.6 µg IDOi per million cells. Correspondingly, the quantified click efficiency was higher than 80% (Figure 2e).

We investigated the stability of SRN‐T cells, especially the retention of SRN on T‐cell surface. The fluorescence intensity of SRN‐RhoB on T‐cell surface gradually decreased over time, nearly a 50% decrease on day 4 post‐fabrication (Figure 2f; Figure S5, Supporting Information). This may be due to that the division and proliferation of T cells led to dilution of SRN.

Next, we investigated the physiological functions of SRN‐T cells. The viability of SRN‐T cells over 10 days was monitored via AnnexinV‐PE‐7ADD apoptosis Kit. As shown in Figure 2g; Figure S6a (Supporting Information), viability of SRN‐T cells was higher that 78% on day 10 post fabrication, showing no significant difference with that of non‐armored T cells (T cells). The proliferation capacity [indicated by the fluorescent intensity of 5(6)‐Carboxyfluorescein diacetate N‐succinimidyl ester (CFSE),^[^ ^28^ ^]^] and activation level [indicated by expression of CD69,^[^ ^29^ ^]^ tumor necrosis factor alpha (TNFα), Interferon gamma (IFNγ),^[^ ^30^ ^]^ and Granzyme B (GzmB)^[^ ^31^ ^]^] of SRN‐T cells were also similar to that of T cells (Figure 2h,i; Figure S6b, Supporting Information). Additionally, we studied the inflammatory tropism of SRN‐T cells. As presented in Figure 2j,k, using chemoattractant monocyte chemoattractant protein‐1 (MCP‐1) as the chemoattractant,^[^ ^32^, ^33^ ^]^ chemotaxic migration of SRN‐T cells followed the concentration gradient of MCP‐1. At the same time, nearly 42% of SRN‐T cells could transmigrate vascular walls (mimicked by intact HUVEC layers) in response to MCP‐1. We believed the above‐mentioned chemotaxic and transvascular migration endowed SRN‐T cells with tumor‐targeting ability. Notably, a certain number of SRN detached from SRN‐T cells during transmigration as indicated by only a half of initial loaded drugs retained on T cells (Figure S7, Supporting Information), which might because deformation of T‐cell membrane during transmigration led to the detachment of lipidic anchor.

Finally, to visualize the spatiotemporally‐controlled behavior of SRN‐T cells, the lipidic anchor and SRN were tagged by FITC and RhoB, respectively. SRN localized well on cell surface at pH 7.4, and detached at pH 6.5 (Figure 2l). Similar to that of SRN, neither of the two drugs was released under physiological conditions from SRN‐T cells (Figure S8, Supporting Information). However, when under acidic conditions (pH 6.5), most of IDOi was released during 12 h, up to 75%. In a medium containing pathological levels of GSH, such as 10 mm, up to ≈70% of CDK4/6i was released during 12 h (Figure 2m). Together, these results demonstrated SRN‐T cells could remain stable under physiological conditions, while spatiotemporally release IDOi in TME and CDK4/6i in tumor cells.

### 2.2 SRN‐T Cells Displayed an Optimized Synergism In Vitro via a Spatiotemporally Controlled Drug Release

**Figure 3:**
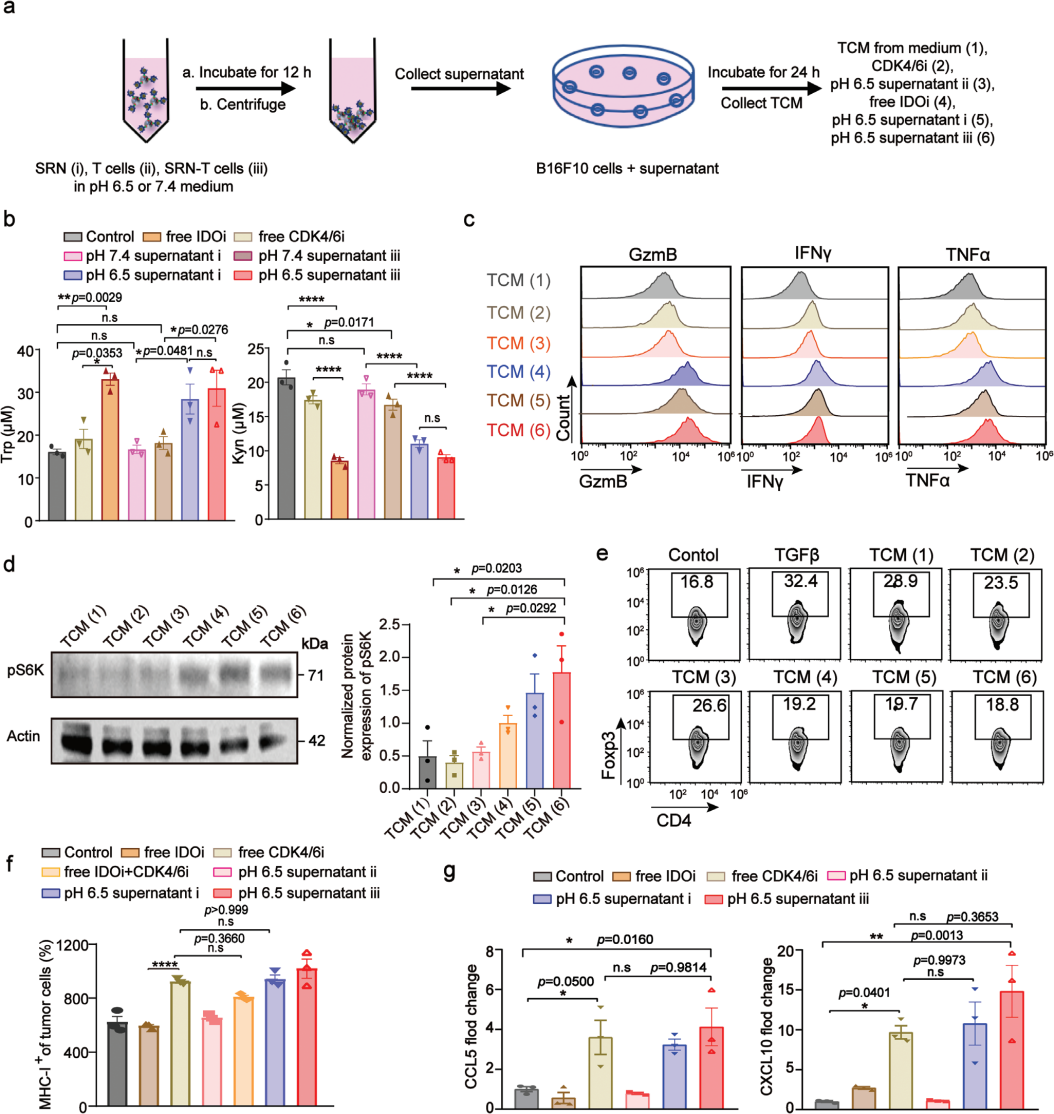
In vitro combinational rationality of spatiotemporally controlled SRN‐T cells. a) Schematic diagram of experimental setup. b) The concentrations of tryptophan (Trp) and kynurenic acid (Kyn) in tumor cells conditioned medium (TCM). c) The expression of activation makers in T cells after treated with different TCM. d) Immunoblotting of downstream mTOR signaling molecules in T cells after treated with different TCM. e) The frequency of Tregs in CD4^+^T cells. f) Histogram of the level of MHC‐I expression of tumor cells after receiving different supernatants. g) Expression level of recruiting chemokines in tumor cells after receiving different supernatants. Data are represented as mean ± SEM; n = 3; ^*^ *p* < 0.05; ^**^ *p* < 0.01; ^****^ *p* < 0.0001; n.s, not significant; determined by one‐way ANOVA with Tukey's correction in b, d, f, g.

The spleen‐derived CD8^+^T cells were isolated from Pmel‐1^+^Thy1.1^+^ transgenic mice, which can target and recognize mouse B16F10 cells. Then, using the isolated CD8^+^T cells to constructed the SRN‐T cells for following experiments. To verify the synergism, we incubated SRN (i), T cells (ii) or SRN‐T cells (iii) in pH 6.5 or 7.4 medium for 12 h, and then supernatant at pH 6.5 or 7.4 were collected and used for following experiments (**Figure** **3**a). As shown in Figure 3b, we found that after treating B16F10 cells with pH 6.5 supernatant i or iii for 24 h, the content of Trp was both improved, accompanied by the decrease of kynurenic acid (Kyn). While, treating B16F10 cells with pH 7.4 supernatant i or iii did not induce a significant change of Trp and Kyn. This might because only at an acidic environment (pH 6.5), could IDOi release from shell of SRN. Next, we examined whether the restored supplement of Trp would promote the activation of T cells. To do so, we collected the tumor cell conditioned media (TCM) from tumor cell receiving: blank medium (1), free CDK4/6i (2), pH 6.5 supernatant ii (3), free IDOi (4), pH 6.5 supernatant i (5), pH 6.5 supernatant iii (6). Then T cells were incubated with collected TCM for pre‐determined time to detect the expression levels of GzmB, IFNγ, and TNFα. As evidenced in Figure 3c, we found that T cells treated with TCM (5) and (6) showed a higher expression of activation makers, comparable to that of TCM (4). Further, we studied the mechanism of how T cell was activated by restored Trp in microenvironment. As shown in Figure 3d and Figure S9 (Supporting Information), T cells treated with TCM (5) and (6) exhibited upregulated phosphorylation levels of S6 kinase (pS6K), a downstream signaling of mammalian target of rapamycin (mTOR).^[^ ^34^, ^35^ ^]^ The results indicated that the restored Trp supplement might active T cells via mTOR pathway, as reported by others.^[^ ^36^ ^]^ In addition to direct activation effect of restored Trp on T cells, we studied whether the restored Trp might suppress proliferation of Treg cells to synergistically improve the activation of effector T cells.^[^ ^37^, ^38^ ^]^ To verify this, we first induced CD4^+^T cells into Treg cells (CD4^+^Foxp3^+^) by transforming growth factor beta (TGFβ).^[^ ^39^ ^]^ Then we treated CD4^+^Foxp3^+^Treg cells with different TCM. As displayed in Figure 3e; Figure S10 (Supporting Information), TCM (5) and (6), containing higher level of Trp, contributed to a lower frequency of Tregs and thus a higher activation of T cells.

Next, we studied how the 1st released liposomal CDK4/6i, interacted with tumor cells to improve T‐cell potency. As displayed in Figure 3f; Figure S11 (Supporting Information), B16F10 cells receiving pH 6.5 supernatant i and iii, showed a significantly up‐regulated expression of MHC‐I, similar to that of B16F10 cells cultured in medium added with equivalent free CDK4/6i. The induced expression of MHC‐I in tumor cells is believed to improve the recognition of tumor cells by T cells, which could further enhance T‐cell activation. Moreover, as shown in Figure 3g, we found tumor cells treated with pH 6.5 supernatant i and iii, displayed a higher expression level of recruiting chemokines, such as CCL5 and CXCL10. This result was in consistent with previous report.^[^ ^40^, ^41^, ^42^ ^]^ The elevated levels of recruiting chemokines have been widely recognized to have a positive relation with enhanced traffic and infiltration of T cells into tumor.^[^ ^43^ ^]^

Encouraged by the superior promoting effect of SRN on T cell, we further investigated the tumor‐killing effects of SRN‐T cells by LDH analysis. As shown in Figure S12 (Supporting Information), when the ration of E: T was at 10: 1, SRN‐T cells displayed the most effective killing capacity toward B16F10 cells.

### 2.3 Tumor‐Tropic Distribution of SRN‐T Cells

**Figure 4:**
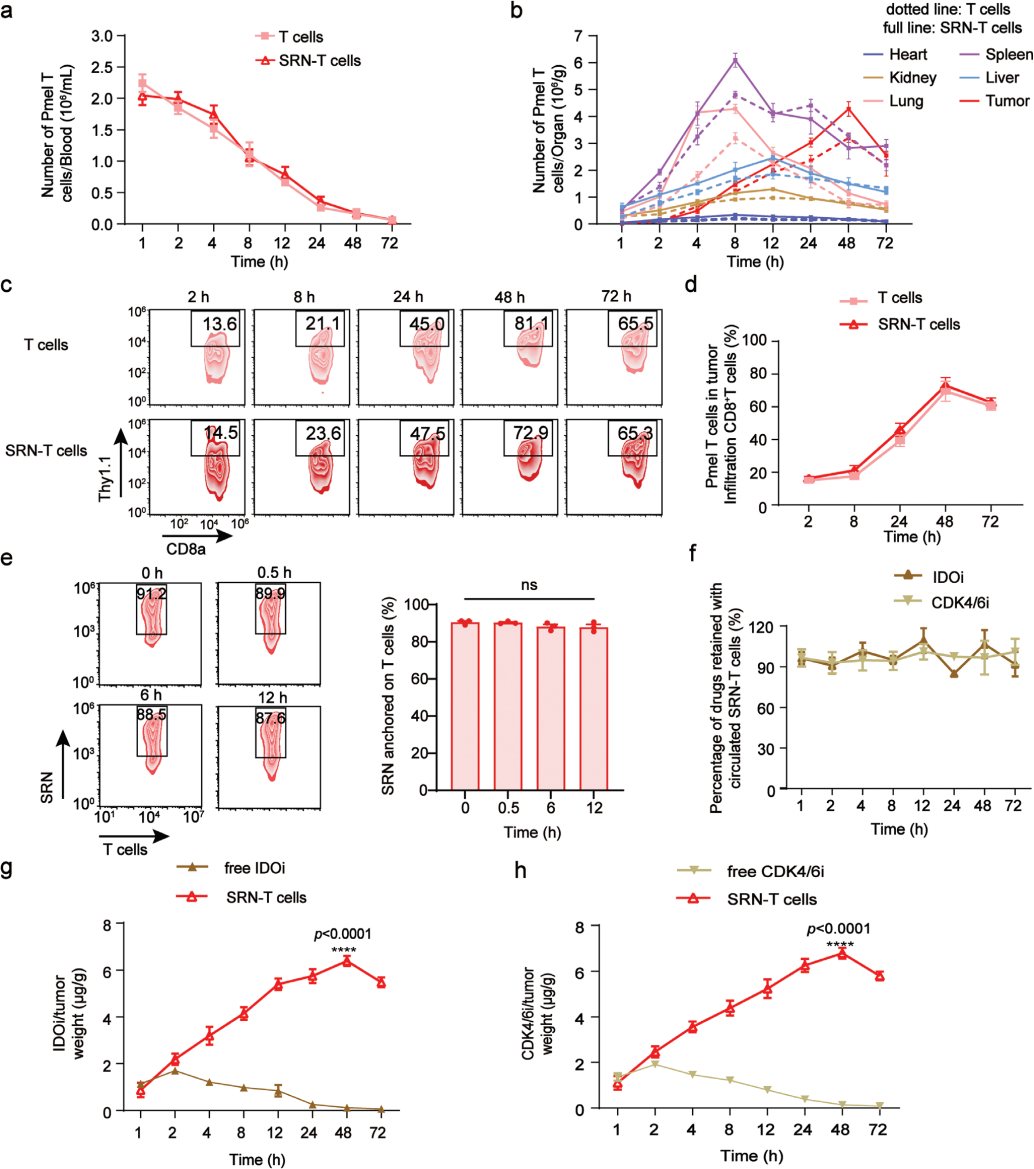
In vivo distribution and stability of SRN‐T cells. a) The amount of SRN‐T cells in the blood circulation after an intravenous transfusion. b) The amount of SRN‐T cells accumulated in major organs and tissues over 3 days. c) Representative flow cytometric analysis and (d) corresponding quantified results of SRN‐T cells in tumor over 3 days. e) Flow cytometric analysis of SRN‐T stability during blood circulation. f) The percentage of drugs remained on SRN‐T cells during blood circulation. The amount of accumulated IDOi g) and CDK4/6i h) in tumor over 3 days. Data are represented as mean ± SEM; n = 3 mice/group; ^****^ *p* < 0.0001; determined by one‐way ANOVA with Tukey's correction in e or unpaired *t* test in g, h.

The amount of SRN‐T cells in blood against post‐*i.v* injection time was first studied. As displayed in **Figure** **4**a, no significant difference was observed between SRN‐T cells and T cells. Then we studied the dynamic accumulation of T cells into major organs and tissues, such as heart, liver, spleen, lung, and kidney. A time‐dependent accumulation mode was observed for both T cells with or without SRN (Figure 4b). Further, we used flow cytometry to investigate the traffic of SRN‐T cells into tumor. Using Thy1.1 as the characteristic marker of transfused T cells, we found that accumulation of SRN‐T cells into tumor peaked at 48 h post transfusion, accounting for nearly 65.3% of total CD8^+^T cells in tumor, which was almost the same to that of T cells (Figure 4c,d), indicating anchorage of SRN did not damage the tumor‐tropic migration of T cells.

The stability of SRN‐T cells during blood circulation was then investigated. To do so, we double labeled SRN‐T cells with CFSE (for T cells) and RhoB (for SRN), which were *i.v*. injected into tumor‐bearing mice. The *i.v*. transfused double‐labeled SRN‐T cells were collected from blood and analyzed by flow cytometry. As displayed in Figure 4e most of transfused SRN‐T cells were stable during blood circulation without significant detachment of SRN. Furthermore, no more than 20% of IDOi and CDK4/6i was detected in blood during circulation of SRN‐T cells as evidenced in Figure 4f demonstrating most of drugs were stably remained on T cells. In contrast, when SRN‐T cells migrated into tumor, SRN was gradually detached due to the acidic microenvironment, as indicated by the separate signals of T cells (green) and SRN (red) in frozen section of harvested tumor (Figure S13, Supporting Information). Then the accumulation of CDK4/6i and IDOi into tumor was studied. As presented in Figure 4g,h, the amount of CDK4/6i and IDOi at tumor sites in mice treated with SRN‐T cells was increased with time, reached the peak level at 48 h post‐injection, significantly higher than those of mice receiving free drugs. This enhanced accumulation of CDK4/6i and IDOi in tumor was believed to be mediated by T‐cell delivery.

### 2.4 Enhanced Anti‐Tumor Efficacy of SRN‐T Cells in a Melanoma‐Bearing Mouse Model

**Figure 5:**
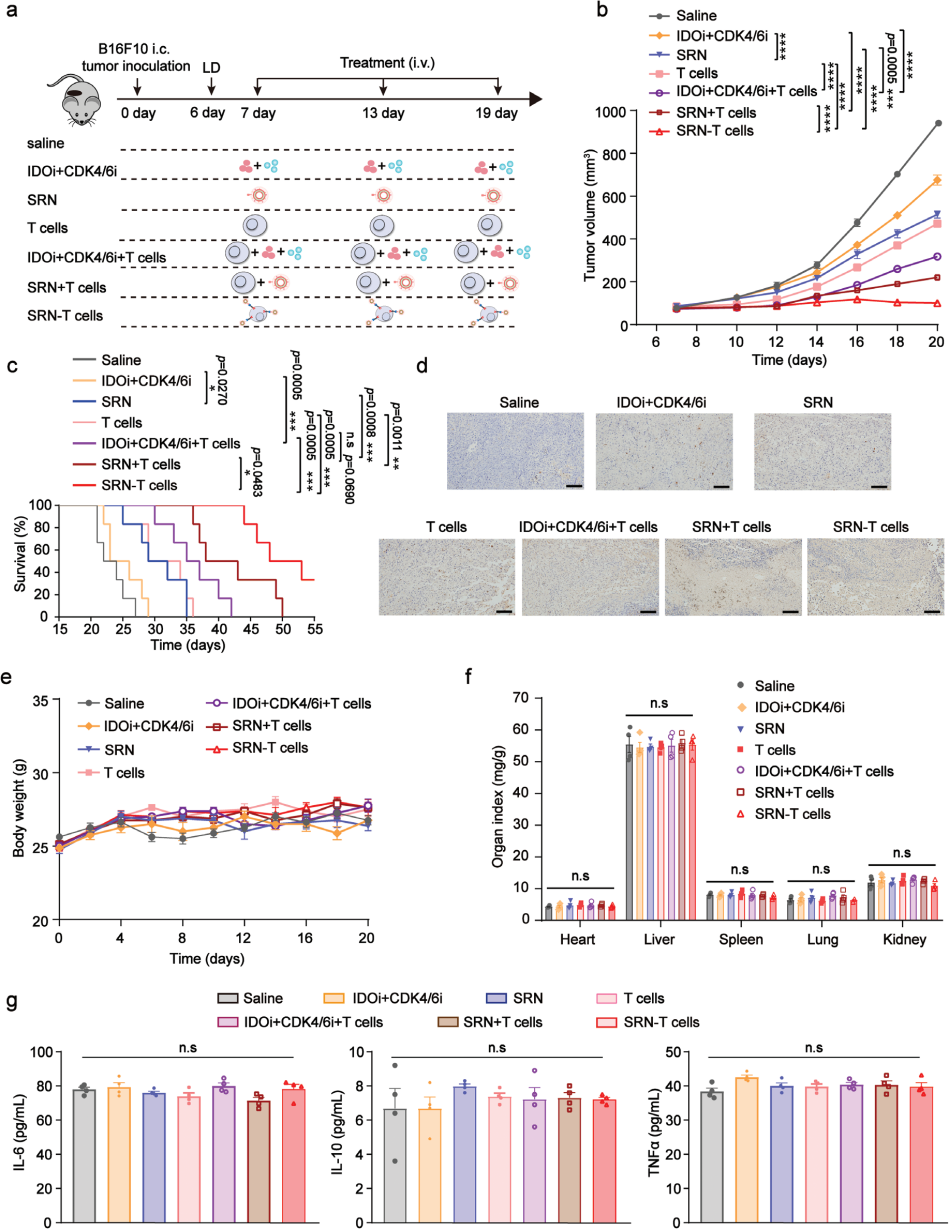
Enhanced anti‐tumor efficacy of SRN‐T cells against melanoma. a) Schematic illustration of the experimental design. *i.v*., intravenous. b) Tumor growth curves of mice *i.v* treated with six doses of indicated formulation (n = 8 mice/group). c) Survival curves of mice receiving six doses of formulation (n = 6 mice/group). d) Histological images of harvested tumors stained by TUNEL. Scale bar: 100 µm. e) Body weights of mice treated with six doses of indicated formulation (n = 8 mice/group). f) Organ indexes of B16F10‐bearing mice receiving six doses of indicated formulation (n = 4 mice/group). g) Serum levels of IL‐6, IL‐10, and TNFα in mice receiving different treatments (n = 4 mice/group). Data are represented as mean ± SEM; ^*^ *p* < 0.05; ^**^ *p* < 0.01; ^***^ *p* < 0.001; ^****^ *p* < 0.0001; n.s, not significant; determined by one‐way ANOVA with Tukey's correction in b, f, g or a log‐rank (Mantel‐Cox) test in c.

The orthotopic melanoma mouse model was established via intradermal injection of B16F10 cells into wild‐type C57BL/6 mice. The mice were randomly divided into seven groups, receiving lymphodepletion on day 6, and intravenously injected with one of the following formulations on Day 7, 13 and 19 post tumor‐implantation: (i) saline, (ii) IDOi+CDK4/6i, (iii) SRN, (iv) T cells, (v) IDOi+CDK4/6i+T cells, (vi) physical mixture of T cell and SRN (SRN+T cells), and (vii) SRN‐T cells (**Figure** **5**a). The tumor volume and body weights of mice receiving different treatments were monitored over 20 days. As exhibited in Figure 5b, tumor of mice treated with SRN‐T cells showed the slowest growth rate and maintained at a very low burden. Correspondingly, after administrated with SRN‐T cells, tumor bearing mice exhibited the longest median survival time, up to 48 days, which was in a stark comparison with saline treated mice‐surviving no more than 27 days (Figure 5c). At therapeutic endpoint, we harvested tumor tissues and analyzed them by TdT‐mediated dUTP Nick‐End Labeling (TUNEL). Tumor tissues collected from mice receiving SRN‐T cells showed the largest brown cell necrotic area, followed by IDOi+CDK4/6i+T cells and SRN+T cells treated groups (Figure 5d, Figure S14, Supporting Information).

To further confirm safety of SRN‐T cells in vivo, we recorded the body weights and organ indexes of experimental mice. As shown in Figure 5e, none of the treatments caused significant loss of body weight over 20 days, suggesting no systemic toxicity being induced in vivo. Consistent with this, the organ indexes of all groups were almost the same, further confirming the safety of SRN‐T cells in vivo (Figure 5f). Moreover, SRN‐T cells showed no harm to the functions of the liver, kidney, or heart [gamma‐glutamyl transferase (GGT), alkaline phosphatase (ALP), aspartate transaminase (AST) and alanine transaminase (ALT) for liver toxicity, lactate dehydrogenase (LDH), blood urea nitrogen (BUN) and creatinine (CRE) for kidney toxicity, α‐hydroxybutyrate dehydrogenase (HBDH) for heart toxicity] (Figure S15, Supporting Information). Furthermore, the histological analysis of the heart, liver, spleen, lung and kidney using the hematoxylin and eosin (H&E) staining evidenced that the treatment with SRN‐T cells displayed no pathological change compared to that with saline (Figure S16, Supporting Information). Additionally, we detected whether the treatment could trigger a systemic inflammatory storm. To do so, we measured the plasma level of Interleukin‐6 (IL‐6), Interleukin‐10 (IL‐10), TNFα in mice receiving different treatments.^[^ ^44^ ^]^ As suggested in Figure 5g, after treated by SRN‐T cells, the plasma level of all cytokines remained almost stable, similar to that of saline‐treated group, which demonstrated the superior in vivo safety of SRN‐T cells.

### 2.5 SRN‐T Cells Augmented T‐Cell Recruitment into Solid Tumors

**Figure 6:**
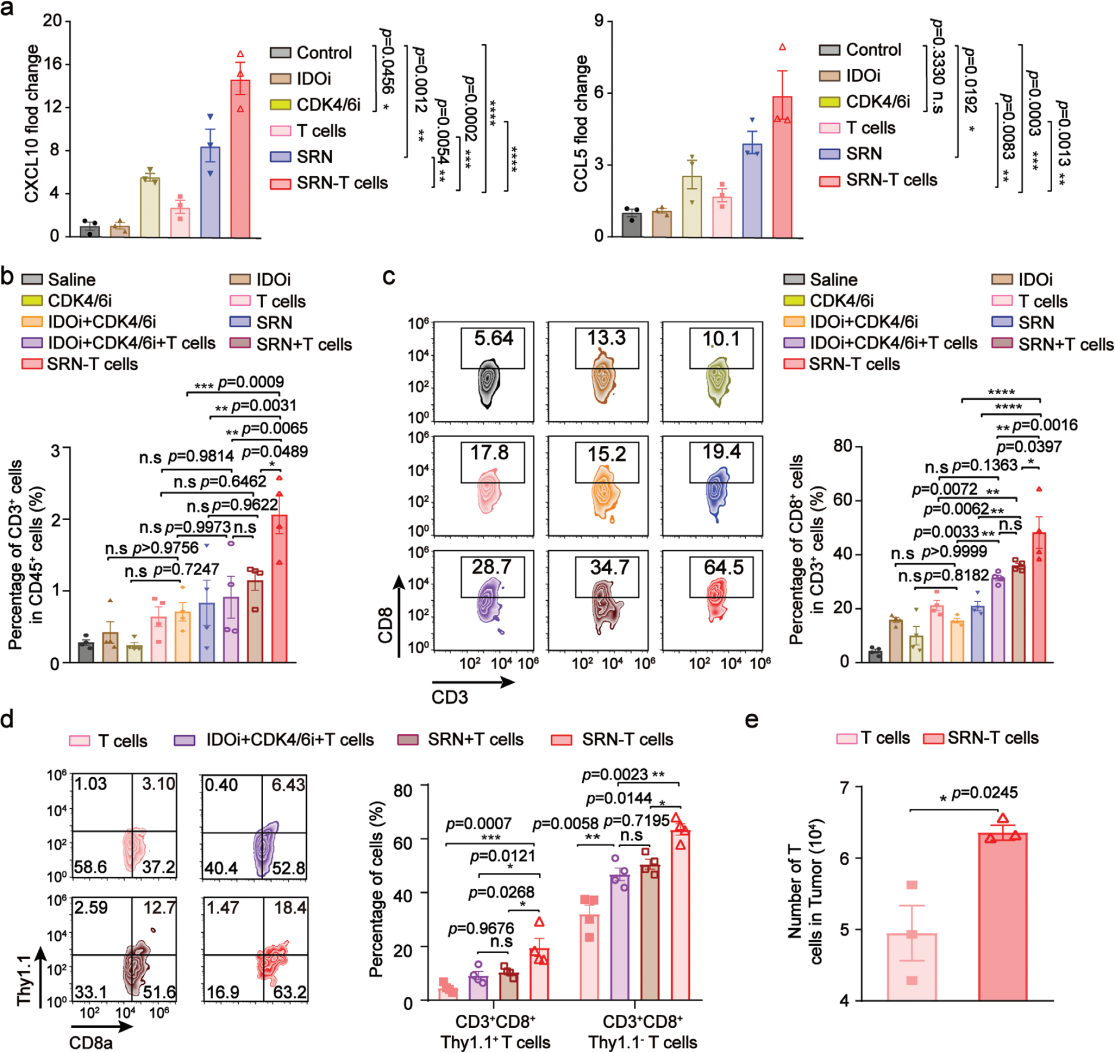
The enhanced recruitment of T cells into tumors was dependent on chemokines. a) Concentration of CCL5 (left) and CXCL10 (right) in tumor harvested from mice receiving different formulations (n = 3 mice/group). b) Percentages of CD3^+^ cells in CD45^+^ cells obtained from tumors treated with different formulations (n = 4 mice/group). c) Representative flow cytometric analysis of CD3^+^CD8^+^T cells (left) and corresponding quantified results (right) in tumor (n = 4 mice/group). d) Representative flow cytometric analysis of CD3^+^CD8^+^Thy1.1^−^ and CD3^+^CD8^+^Thy1.1^+^T cells in tumor, and corresponding quantified results (n = 4 mice/group). e) The amount of T cells with or without SRNs accumulated in tumor (n = 3 mice/group). Data are represented as mean ± SEM; ^*^ *p* < 0.05; ^**^ *p* < 0.01; ^***^ *p* < 0.001; ^****^ *p* < 0.0001; n.s, not significant; determined by one‐way ANOVA with Tukey's correction in a, b, c, d or unpaired *t‐*test in e.

The concentration of recruiting chemokines, e.g. CCL5 and CXCL10, in tumor after administration of different formulations, were studied at the end of treatments (19 days). As displayed in **Figure** **6**a, levels of CCL5 and CXCL10 were significantly increased by 5 and 12 times, respectively in tumors from SRN‐T cells‐treated mice as compared to that of Saline‐treated mice. Then we conducted flow cytometry immunophenotyping to determine the effect of increased chemokines on the composition of tumor immune microenvironment, especially T cells. The percentage of tumor‐infiltrating CD3^+^T lymphocytes were significantly increased from SRN‐T cells‐treated mice as compared to that of other treatments (Figure 6b). Meanwhile, CD3^+^CD8^+^T cells accumulated the most in SRN‐T cells‐treated tumors (Figure 6c). We then studied whether the frequency of endogenous (CD3^+^CD8^+^Thy1.1^−^) or exogenously transferred (CD3^+^CD8^+^Thy1.1^+^) T cells in tumor was both improved. As evidenced in Figure 6d, SRN‐T cells‐treated tumors displayed the largest proportion for both endogenous (CD3^+^CD8^+^Thy1.1^−^) and exogenously transferred (CD3^+^CD8^+^Thy1.1^+^) T cells, measured at 63.2% and 18.4%. While, the percentages of CD3^+^CD8^+^Thy1.1^−^ and CD3^+^CD8^+^Thy1.1^+^ T cells in mice administrated with SRN+T cells were measured at 51.6% and 12.7%, respectively. In a stark comparison, the frequency of CD3^+^CD8^+^Thy1.1^−^ and CD3^+^CD8^+^Thy1.1^+^ T cells in mice receiving T cell was quantified at 37.2% and 3.10%, respectively (Figure 6d). Taking together, we believed by elevating the expression of recruiting chemokines, especially CCL5 and CXCL10, by tumor cells, the spatiotemporally‐ruptured SRN boosted the quantity of both exogenously transfused and endogenous CD8^+^T cells. Lastly, the number of exogenously transferred T cells in tumor were quantified. We found that although there was no significant difference in tumor‐tropic capacity of T cells with and without SRNs (Figure 2k and Figure 4c), the overall accumulation of SRN‐T cells after three injections increased by ≈0.3 times (Figure 6e), which might because the positive recruiting feedback loop established by SRNs took time.

### 2.6 SRN‐T Cells Boosted T‐Cell Activation in Tumor

**Figure 7:**
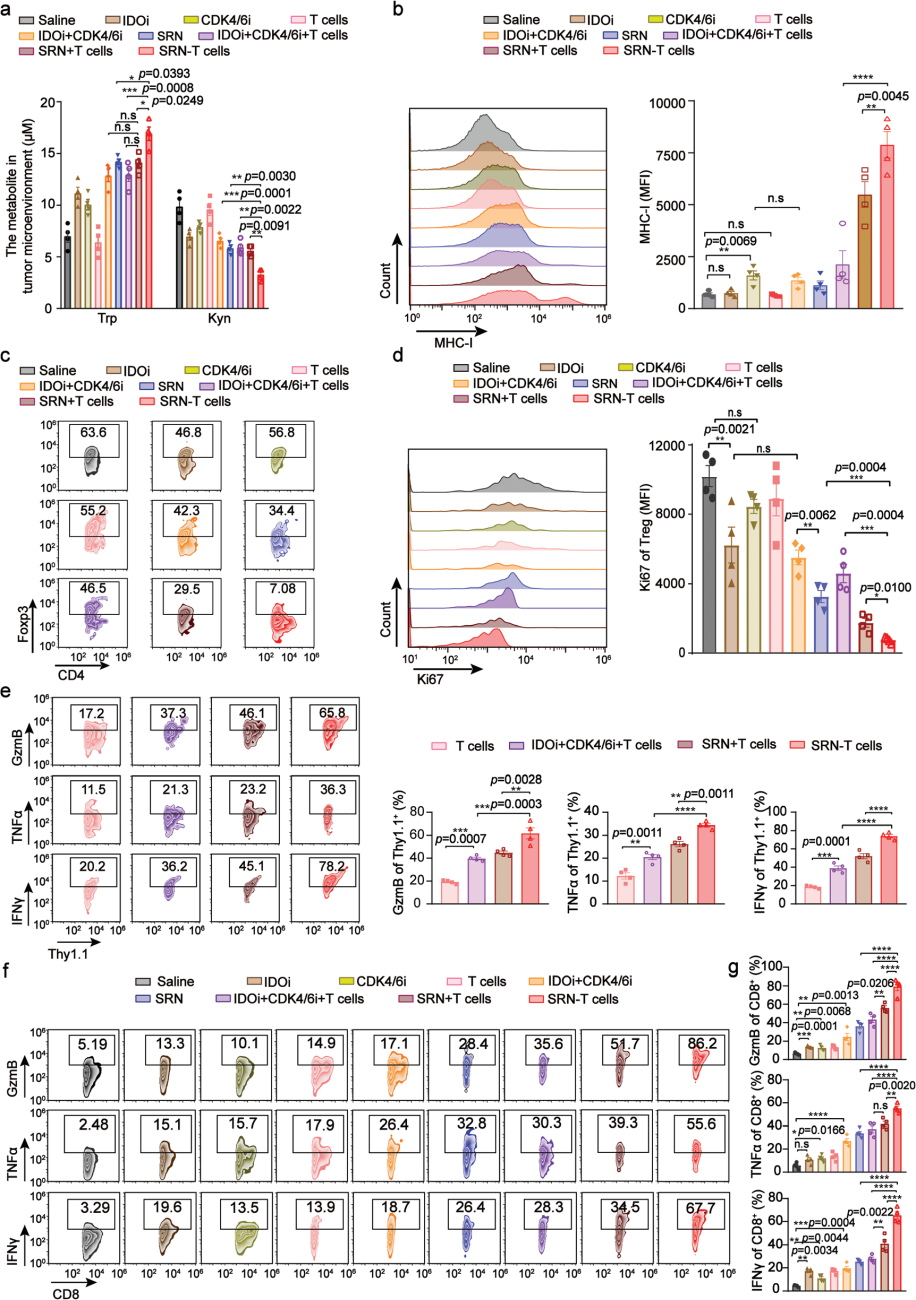
SRN‐T cells restored nutrient supply and induced MCH‐I expression to improve the activation of T cells a) The level of Trp and Kyn in tumor after administration of different formulations. b) Representative flow cytometric analysis of the level of MHC‐I expression in B16F10 cells (left), and corresponding quantified results (right). c) Representative flow cytometric analysis of frequency of Tregs (CD4^+^Foxp3^+^T cells) in tumors harvested from mice treated with different formulations. d) Representative flow cytometric analysis of the count of Ki67^+^ Tregs (left), and corresponding quantified results (right). e) Representative flow cytometric analysis of CD8^+^Thy1.1^+^IFNγ^+^, CD8^+^Thy1.1^+^TNFα^+^ and CD8^+^Thy1.1^+^GzmB^+^ Tcells in tumors obtained from mice receiving different treatments (left) and corresponding quantified results (right). f) Representative flow cytometric analysis of CD8^+^IFNγ^+^, CD8^+^TNFα^+^ and CD8^+^GzmB^+^ T cells in tumors obtained from mice receiving different treatments and corresponding quantified results (g). Data are represented as mean ± SEM; n = 4 mice/group; *P < 0.05; **P < 0.01; ***P < 0.001; ****P < 0.0001; n.s, not significant; determined by one‐way ANOVA with Tukey's correction in a, b, d, e, g.

We measured the level of Trp and Kyn in tumor after administration of different formulations. A significant increase of Trp and decrease of Kyn were both observed in tumor receiving SRN‐T cells, compared to those of other groups (**Figure** **7**a). The expression of MHC‐I of tumor cells were also examined by flow cytometry. Tumors of mice receiving SRN‐T cells showed the most expression of MHC‐I (Figure 7b). We believed the increased level of Trp and MHC‐I in microenvironment was due likely to the sequential release of IDOi and CDK4/6i in tumor mediated by SRN (Figure 4). Then we examined the proliferation status of Tregs in tumor. As suggested in Figure 7c,d; Figure S17 (Supporting Information), SRN‐T cells treated tumor consisted of least frequency of Tregs, nearly 7.08%, as well as the lowest expression of Ki67. We proposed the increased level of Trp and MHC‐I as well as the suppressed proliferation of Tregs in tumor microenvironment would contribute to an enhanced activation of effector T cells.

To verify this, tumor‐infiltrating CD8^+^T cells (including endogenous and transfused exogenous ones) were isolated, of which activation marks, including IFNγ, TNFα, and GzmB, were detected by flow cytometry. We found that the quality of exogenously transfused T cells (CD8^+^Thy1.1^+^), was significantly improved because of augmenting effect mediated by SRN. The activated phenotype proportions were measured at 65.8%, 36.3%, and 78.2% for GzmB, TNFα, and IFNγ, respectively, which were followed by groups treated by SRN+T cells. While, IDOi+CDK4/6i+T cells treated tumors consisted of moderate proportion of activated T cells (Figure 7e). In a stark contrast, tumors harvested from mice receiving T cells consisted of least amount of activated CD8^+^T cells. It should be noted that, the overall quality of tumor‐infiltrating CD8^+^T cells (including endogenous and transfused exogenous ones) displayed a favorable enhancement (Figure 7f,g).

## 3 Conclusion

Given the various therapeutic challenges of solid tumors, including nutrient starvation, limited level of recruiting chemokines, etc,^[^ ^45^, ^46^, ^47^ ^]^ we developed a triple drug combination for T‐cells mediated solid tumor immunotherapy. Concretely speaking, IDOi, e.g. indoximod, is an increasingly validated class of potential therapeutics for improving T‐cells activation by lowering the rapid consumption of Trp by tumor cell.^[^ ^48^ ^]^ The restored level of Trp in TME could activate T cells by upregulating the phosphorylation level of S6K, a downstream signal of the mTOR pathway, along with decreasing the proliferation of Tregs in tumor.^[^ ^37^, ^38^ ^]^ The combination of T cells with IDOi has shown improved therapeutic outcome in several pre‐clinical tumor models.^[^ ^49^, ^50^, ^51^ ^]^ However, the clinical benefits of this two‐drug combination were limited, which might be owing to the poor infiltration of T cells in most solid tumors.^[^ ^52^ ^]^ Therefore, in this study, we combined CDK4/6i with T cells and IDOi, to treat melanoma‐a T cell excluded “cold” tumor.^[^ ^53^ ^]^ Since CDK4/6i is reported to promote recruitment of T cells into mammary tumors by inducing expression of T cell chemotactic chemokines in tumor cells, What's more, CDK4/6i can induce the expression of MHC‐I in tumor cells to break immunoescape.^[^ ^54^ ^]^

To achieve an optimized synergism among this trip‐drug combination, we established a spatiotemporally‐controlled cell‐engineering technology to formulate these triple drugs into one cell therapeutic (SRN‐T cells). A spatiotemporally‐ruptured core‐shell nanoparticle (SRN) containing both IDOi and CDK4/6i, were first fabricated and clicked onto T cells to yield SRN‐T cells. In a pre‐clinical mouse model of melanoma, we demonstrated the rationality of this triple combination. Moreover, the super anti‐tumor capacity of this novel SRN‐T cells, was also displayed. Additionally, we uncovered the mechanism of the superior anti‐tumor efficiency was due to augmented recruitment and activation of T cells, which was mediated by the site‐specific stimuli‐activated drug release behavior of SRN‐T cells. That is : i) shell encapsulating IDOi was ruptured in response to acidic pH in TME, contributing to 1st release of IDOi^,^ and consequently enhancing the activation of T cells (Figures 3a‐e and 7), and then ii) GSH‐sensitive core loading CDK4/6i was internalized by tumor cells, and achieved a 2nd release of CDK4/6i, leading to an up‐regulated expression of inflammatory chemokines as well as MHC‐I, and thus an increased infiltration and activation of T cells in tumor (Figures 3f,g and 6).

In conclusion, with an eye on the multidimensional complexity of solid tumor, we developed a tri‐drug combination of T cells, IDOi and CDK4/6i, for solid tumor T‐cell immunotherapy. To optimize the synergism, we established a spatiotemporally controlled cell engineering technology to formulate triple drugs into one novel cell therapeutic, namely SRN‐T cells. The SRN‐T cells actively migrated into tumor sites, and unloaded the drugs in a spatiotemporally controlled manner to achieve a significantly improved anti‐tumor efficiency. Our study demonstrated the rationality and superiority of a novel tri‐drug combination mediated by the spatiotemporally controlled cell‐engineering technology, which provides new insights into solid tumor immunotherapy.

## 4 Experimental Section

### Cell Lines and Mice

The mouse melanoma cell line B16F10 was purchased from the American Type Culture Collection and was cultured in DMEM with 10% FBS and 1% Penicillin‐Streptomycin. TCR transgenic Thy1.1^+^ pmel‐1 mice were purchased from Jackson Laboratory. C57BL/6J mice (18‐20 g, male) were obtained from Beijing Vital River Laboratory Animal Technology Co. Ltd (Beijing, China). All the animals were pathogen free and allowed access to food and water freely. The Animal Care and Use Committee of the China Pharmaceutical University approved all of the animal studies and animal protocols (Approval Number: 2020‐08‐007).

### Preparation and Characterization of SRN‐T Cells

SRN‐T cells were obtained by incubating CD8^+^T cells sequentially with DPPE‐PEG_5K_‐N_3_ and SRN. Briefly, CD8^+^T cells (1 × 10^6^ cells mL^−1^) were resuspended in serum‐free medium. Then, CD8^+^T cells were incubated with 500 µL DPPE‐PEG_5K_‐N_3_ (20 µm) solution at 37 °C for 10 min. The cells were collected, washed twice with PBS, and centrifuged. The cell precipitation was then resuspended with 1 mL SRN solution containing 150 µg mL^−1^ CDK4/6i and incubated at 37 °C for 45 min. The cells were collected, and washed twice with PBS.

For the preparation of RhoB‐labelled SRN‐T cells, CD8^+^T cells (1 × 10^6^ cells mL^−1^) were incubated with 500 µL DPPE‐PEG_5K_‐N_3_ (20 µm) or DPPE‐PEG_5K_ (20 µm) solution at 37 °C for 10 min. After washing with PBS, SRN‐RhoB were incubated with cells at 37 °C for 45 min. After washing with PBS, the cells were stained with Hoechst 33 342, fixed with 4% PFA, and used immediately for the confocal imaging and flow cytometry analysis, the untreated T cells as control. To investigate the stability of SRN on the surface of T cells, RhoB‐labelled SRN‐T cells were cultured and expanded for another 2 and 4 days. Then, the cells were collected and fixed with 4% PFA for flow cytometry analysis.

To investigate the drug release of SRN‐T cells, the RhoB‐labelled SRN‐T cells were incubated with the medium of pH 7.4 or pH 6.5, after incubation for 4 h, the cells were collected and stained with Hoechst 33 342, fixed with 4% PFA, and used immediately for the confocal imaging. The SRN‐T cells were incubated with the medium of pH 7.4 or pH 6.5, after incubation for different time (0, 1, 2, 4, 8, and 12 h), the supernatants were collected and measured by HPLC. Moreover, SRN‐T cells were incubated with normal blood environment (pH 7.4, containing 0.01 mm GSH), tumor tissue microenvironment (pH 6.5, containing 2 mm GSH), and tumor cell cytosolic environment (pH 6.5, containing 10 mM GSH), respectively. After incubated for different time (0, 1, 2, 4, 8, and 12 h), the supernatants were measured by HPLC. Then, the release amounts of CDK4/6i and IDOi at different time were calculated and the release profiles were plotted.

### Evaluation of Physiological Functions of SRN‐T Cells

For the apoptosis analysis, 1×10^6^ T cells and SRN‐T cells after 10‐day stimulation were collected and suspended in 0.5 mL of 1×binding buffer and washed twice with ice‐cold PBS. The Annexin V‐PE Apoptosis Detection Kit (Vazyme, China) was used according to the manufacturer's instructions for the apoptosis assay. The stained cells were analyzed by the Attune NxT flow cytometer (Thermo).

For the proliferation assay, T cells and SRN‐T cells were resuspended at 1×10^7^/mL in pre‐warmed serum‐free RPMI. CFSE was added to the cells at a final concentration of 2 µm, which were incubated at 37 °C for 15 min. Then, the cells were washed with the cold RPMI medium with 10% FBS (1: 1, *v: v*) twice to quench the CFSE staining. Then the cells were cultured in RPMI medium containing 10 ng/mL recombinant mouse IL‐2 in the culture plate pre‐coated with 5 µg mL^−1^ anti‐CD3 and 2 µg mL^−1^ anti‐CD28 agonist antibodies. After activated for 24 h, the cells were collected at day 2 and day 4, and washed with PBS before analysis by flow cytometry. Meanwhile, T cells and SRN‐T cells were cultured in RPMI medium containing 10 ng mL^−1^ recombinant mouse IL‐2 in the culture plate pre‐coated with 5 µg mL^−1^ anti‐CD3 and 2 µg mL^−1^ anti‐CD28 agonist antibodies. At day 0, 3, 6, 8, and 10, the cells were collected and counted through the cell counter. Fold expansions rate (%) = Number of cells/Number of cells (day 0) × 100%.

To demonstrate the activation of T cells, T cells or SRN‐T cells were plated on 96 well microtiter plates and stimulated with or without anti‐CD3 and anti‐CD28 agonist antibodies. After 24 h incubation, the cells were collected and stained with FITC anti‐mouse CD69 antibody (Biolegend), and the expression levels of the early T cell activation markers CD69 were quantified by flow cytometry. Moreover, T cells or SRN‐T cells were stimulated with concanavalin A (ConA, Sigma) for 2 days and rested for another 1 day. Then the cells were collected and stained with PE/Cyanine 7 anti‐mouse GZMB antibody, Brilliant Violet 510 anti‐mouse IFNγ antibody, and PE anti‐mouse TNFα antibody. After washed with PBS, the cells were measured by flow cytometry.

The chemotaxis of SRN‐T cells was investigated using a transwell migration assay (Corning). Briefly, 1×10^6^ SRN‐T cells or unmodified T cells were added to the upper chamber of the transwell, and different concentrations of MCP‐1 (5 ng/mL, 20 ng/mL, 100 ng/mL) was added into the lower chamber to induce the migration. After 12 h of incubation, the cells in the lower chamber were harvested and the numbers were counted using a hemacytometer. The chemotaxis index [(N_SRN‐T cells_ – N_control_)/N_control_)] was calculated, where N_SRN‐T cells_ and N_control_ are the counted numbers of CD8^+^T cells in the lower chamber after incubating with SRN‐T cells in the presence of MCP‐1 and the T cells in the absence of MCP‐1, respectively.

Chemotactic migration across vascular endothelial cells. A transwell model with a confluent endothelial (HUVEC) monolayer was established to investigate the transvascular capacity of SRN‐T cells. Briefly, HUVECs (2×10^5^ cells/well) were seeded onto the upper chamber of the transwell (3 µm, 24 mm) and cultured with the medium containing FBS (10%, *v: v*). The integrity of the cell monolayer was evaluated by measuring the transepithelial electrical resistance (TEER) values using a Millicell‐ERS voltohmmeter (Millipore). The cell monolayers with TEER value higher than 300 Ω cm^2^ were used for the transmigration studies. SRN‐T cells and T cells were added to the upper chamber with incubation of 25 ng mL^−1^ TNFα for 4 h respectively, and 20 ng mL^−1^ MCP‐1 was added into the lower chamber to induce the migration. After 12 h of incubation, the medium in the upper chamber and the lower chamber were collected. The amount of CDK4/6i and IDOi in the supernatant and intracellular were determined using HPLC. The CDK4/6i and IDOi ratio in each compartment was calculated compared with the feeding amount of CDK4/6i and IDOi. The number of T cells in the lower chamber was also counted.

### The Optimized Synergism of SRN‐T cells In Vitro

SRN (4.8 µg mL^−1^, i), T cells (5 × 10^5^ mL^−1^, ii), SRN‐T cells (5 × 10^5^ mL^−1^, iii) were incubated in medium (pH 6.5 or pH 7.4) for 12 h and the supernatant was collected, respectively. Meanwhile, B16F10 cells (5 × 10^5^ cells mL^−1^) were plated in a 96‐well plate with 200 µL per well, after the cells were attached to the wall, the supernatant was discarded and washed with PBS. Each well was added with 200 µL above supernatant (i, iii) and continued incubation to obtain tumor conditions medium (TCM).

B16F10 cells was plated into a 96‐well plate, after the cells were attached to the wall, the supernatant was discarded and washed with PBS. Then, the wells were added with 200 µL different medium, including RPMI medium, free IDOi (4.6 µg/mL), free CDK4/6i (4.8 µg/mL), group i, and iii in medium (pH 6.5 or pH 7.4), respectively. After cultured for 24 h, the contents of tryptophan and kynurenine in the supernatant of each group were detected by ELISA kit.

TCM coming from tumor cells treated with medium (1), CDK4/6i (2), PH 6.5 supernatant ii (3), free IDOi (4), pH 6.5 supernatant i (5), PH 6.5 supernatant iii (6) was collected and co‐incubated with CD8^+^T cells (2 × 10^6^ cells/mL) for 48 h. Then, CD8^+^T cells were collected and suspended in RPMI 1640 with 1 mL PMA (100 ng/mL), Ionomycin (2 µM), and BFA (10 µg/mL) for 4 h. After washed with PBS, the cells were fixed with 4% PFA for 10 min, and perforated with 0.1% Triton for 10 min. Then, the cells were stained with PBS solution containing PE/Cyanine 7 anti‐mouse GZMB antibody (0.8 µg/mL, Biolegend), Brilliant Violet 510 anti‐mouse IFNγ antibody (0.8 µg/mL, Biolegend) and PE anti‐mouse TNFα antibody (0.8 µg/mL, Biolegend) for 30 min. Pre‐cooling PBS washing twice. Fluorescence intensity was measured by flow cytometry.

Meanwhile, the above CD8^+^T cells was also collected by dealt with the aforementioned way. Then, 120 mL SDS lysate (mixed with protease inhibitor, *v: v* = 1:100) was added into each tube, eddied and placed in the ice bath for full lysis for 20 min. After pyrolysis, the supernatant was centrifuged at 12 000 rpm for 10 min, and part of the supernatant was taken for BCA (Bicinchoninic acid) protein quantification. The remaining supernatant was supplemented with 4× protein load buffer, beaten and mixed, and cooked in a metal bath at 100 °C for 10 min to fully denature the protein. Samples were stored at −80 °C for later use. Then, the protein was loaded, and the amount of protein was 20 µL per well. The electrophoresis procedure was set as follows: constant pressure, 100 V, 90 min. After electrophoresis, PVDF membrane (0.2 µm) was activated with methanol for 3 min, followed by membrane transfer operation. The film transfer procedure was set as follows: constant current, 300 mA, 90 min. After the membrane transfer, the membrane was immersed in 5% skimmed milk powder solution and closed at 37 °C by a constant temperature shaker for 1 h. Then, the membrane was rinsed with TBST solution (TBS buffer containing 0.1% Tween‐20) for 3 times, 5 min each time. Cut the film to a suitable strip, immerse the strip in PS6K antibody solution (5% BSA solution, *v: v* = 1:1000), and incubate overnight at 4 °C; The PS6K antibody solution was recovered, and the strips were rinsed with TBST solution for 3 times, 5 min each. Then, the strips were immersed in goat anti‐rabbit secondary antibody solution (5% skimmed milk powder solution, *v: v* = 1:10000) and incubated at 37 °C for 1 h. After incubation, the strips were rinsed with TBST solution for 3 times, 5 min for each time. Finally, Electrochemiluminescence (ECL) luminescence liquid is added to the strips by automatic fluorescence/chemiluminescence imaging system.

CD4^+^T cells (4 ×10^5^) were incubation with different medium as following: 1) RPMI medium; 2) 5 ng/mL TGFβ; 3–8) was TCM coming from tumor cells treated with medium (1), CDK4/6i (2), PH 6.5 supernatant ii (3), free IDOi (4), pH 6.5 supernatant i (5), PH 6.5 supernatant iii (6), respectively. After incubation for 48 h, the cells in each group were collected and stained with PE anti‐mouse Foxp3 antibody (Biolegend) and Brilliant Violet 421 anti‐mouse CD4 antibody (Biolegend) to detect the expression of Foxp3 in CD4^+^T cells by flow cytometry.

B16F10 cells (5×10^5^ cells /mL) were plated at six‐well plate. After the cells adhered to the wall, the supernatant was discarded and washed with PBS. Then, different medium (1 mL) was added including: free CDK4/6i (4.8 µg/mL), free IDOi (4.6 µg/mL), free IDOi (4.6 µg/mL) +CDK4/6i (4.8 µg/mL), pH 6.5 supernatant ii (1×10^6^ cells), pH 6.5 supernatant i (4.8 µg/mL) and pH 6.5 supernatant iii (1×10^6^ cells). After incubation for 48 h, the supernatant was collected and washed with PBS. After digestion with trypsin, the B16F10 cells were collected and stained with APC anti‐mouse MHC‐I antibody (0.6 µg/mL, Biolegend) in PBS solution for 30 min. Then, the cells were pre‐cooled and washed twice with PBS. The expression of MHC‐I in B16F10 cells was determined by flow cytometry after being fixed at room temperature for 10 min with 4% PFA and washed with PBS. Meanwhile, the supernatant was collected to evaluated the expression of the recruiting chemokines (CCL5 and CXCL10) by ELISA kit followed manufacturer's manual.

To measure CD8^+^T‐cell cytotoxicity, activated CD8^+^T cells, CDK4/6i and IDOi pretreated CD8^+^T cells and SRN‐T cells were washed three times with PBS, then the eight groups of cells were mixed with B16F10 (1×10^5^) in the medium (phenol‐free RPMI 1640, 2% FBS) at the ratios of 10:1, respectively. After 24 h and 48 h, the cytotoxic efficiency was measured by quantifying the release of endogenous lactate dehydrogenase (LDH) from B16F10 cells using LDH Cytotoxicity Assay Kit.

### In Vivo Distribution of SRN‐T Cells

B16F10 cells in logarithmic growth phase were selected, and the cell density was adjusted to 1×10^7^ cells /mL by PBS. A density of 1×10^6^ cells/mouse was inoculated under the right forearm of 20–22 g male C57BL/6J mice and reared in a clean grade feeding room. Tumor diameter was measured with a vernier caliper, and the tumor volume was calculated according to the following formula: V = L×W×W/2, where L is the long diameter of the tumor and W is the short diameter of the tumor. When the volume reaches 50 mm^3^, it is used for in vivo experiments.

The tumor‐bearing mice were given T cells or SRN‐T cells, after injection for different time the blood was collected from orbital, and the mice were euthanized. Then, the organ including heart, liver, spleen, lung, kidney and tumor was collected. The blood was centrifugated and the cells were lysed of red blood cells and isolated by 40 to 70% Percoll (GE) gradient centrifugation to obtain lymphocytes. The lymphocytes were stained with Brilliant Violet 421 anti‐mouse Thy1.1 antibody (0.5 µg/mL, Biolegend) and APC/Cyanine7 anti‐mouse CD8a antibody (1 µg/mL, BD) for 30 min, and washed twice with pre‐cooled PBS for detection of flow cytometry. These organs were weighted and digested by collagenase IV (Sangon Biotech) to obtain a single‐cell suspension. Then, the lymphocyte was isolated by 40 to 70% Percoll (GE) gradient centrifugation and analyzed by flow cytometry to determine the quantitation of T cells in organs.

Tumor‐bearing mice were administrated with SRN‐T cells, in which SRNs were labelled by Rhodamine B and T cells were labelled by CFSE. The blood was collected at post‐injection 0, 0.5, 6, and 12 h, which was then centrifuged (300 g, 10 min). The obtained pellets were dispersed into Red Blood Cell Lysis Buffer to get rid of red blood cells, and then isolated by Percoll density gradient centrifugation. The yielded lymphocytes were then analyzed by flow cytometry. Meanwhile, the harvested tumors at post‐injection 0, 0.5, 6, 12, 24, 48 h, was cryosectioned, stained by 0.1% Hoechst33342, and subsequently imaged by Panoramic tissue slice scanner (NanoZoomer S60 C13210‐01).

The tumor‐bearing mice were given free IDOi, free CDK4/6i or SRN‐T cells, respectively. After injection for different time the blood was collected from orbital, and the mice were euthanized. Then, the tumors were collected. The blood was centrifugated and the serum was collected. Then, the serum was added protein precipitator (acetonitrile: methanol = 2:1), followed by vortex for 5 min and centrifugation at 12 000 rpm for 10 min. The supernatant was analyzed by HPLC to determine the concentration of IDOi and CDK4/6i in blood. Moreover, the tumors were weighted and homogenized in saline, the homogenate was diluted with 10% NaHCO_3_ aqueous solution and mixed with acetonitrile, followed by vortex for 5 min and centrifugation at 12 000 rpm for 10 min. Then, the supernatant was quantified by HPLC to determine the concentration of IDOi and CDK4/6i in tumor.

### In Vivo Anti‐Tumor and Optimized Synergism of SRN‐T cells

: The tumor‐bearing mice were lymphodepleted with fludarabine (2 mg kg^−1^) and cyclophosphamide (2 mg kg^−1^) on day 6. Then, the mice were randomly divided into 7 groups with 8 mice in each group and received: 1) saline; 2) CDK4/6i (4 mg kg^−1^) + IDOi (3.5 mg kg^−1^); 3) SRN (4 mg kg^−1^, CDK4/6i concentration as reference); 4) T cells (1.5×10^7^ cells/mouse); 5) IDOi (3.5 mg kg^−1^) + CDK4/6i (4 mg kg^−1^) + T cells (1.5×10^7^ cells/mouse); 6) SRN (4 mg kg^−1^, based on CDK4/6i quality) + T cells (1.5×10^7^ cells/mouse); 7) SRN‐T cells (1.5×10^7^ cells/mouse) by intravenous injection on days 7, 13 and 19 after tumor inoculation, respectively.

On day 7, 10, 12, 14, 16, 18, and 20 after tumor bearing, the long diameter (L) and short diameter (W) of tumor bearing mice were measured, respectively. The tumor volumes were calculated according to the formula V = L×W×W/2, and the tumor volume‐time curve (tumor inhibition curve) was plotted. The survival status of the mice was observed every day after tumor bearing, and the death cases of mice were counted when the mice occurred natural death or their tumor volume reached 1500 mm^3^ which would be euthanized. The survival curve of the mice was finally statistically derived according to the survival status of the mice. Tumor tissues isolated from tumor‐bearing mice were divided into paraffin‐embedded sections, and the tumor tissues were stained with TUNEL (TDT‐mediated DUTP Nick End Labeling). The tumor tissues were imaged under positive fluorescence microscope, and the apoptosis and proliferation of each group of tumors were observed by photography.

The body weight of mice was recorded on day 2, 4, 6, 8, 10, 12, 14, 16, 18, and 20, respectively. On day 21 after tumor bearing, four mice from each group were randomly selected, and the hearts, livers, spleens, lungs, and kidneys of the obtained mice were stripped and weighed after euthanasia, then the organ/body weight ratio was calculated in each administration group. Serum samples from each group of mice were collected at day 18 after tumor inoculation and these were then analyzed for cytokine levels of IL‐6, IL‐10, and TNFα using the ELISA Kit (Elabscience, China).

When the tumor volume of C57BL/6J mice reached 50 mm^3^, the tumor‐grafted mice were randomly divided into 9 groups with 4 mice in each group and given: 1) saline; 2) IDOi (3.5 mg kg^−1^); 3) CDK4/6i (4 mg kg^−1^‐); 4) IDOi (3.5 mg kg^−1^‐)+CDK4/6i (4 mg kg^−1^); 5) SRN (4 mg kg^−1^‐, taking CDK4/6i content as reference); 6) T cells (1.5×10^7^ cells/mouse); 7) IDOi (3.5 mg kg^−1^) + CDK4/6i (4 mg kg^−1^‐) + T cells (1.5×10^7^ cells/mouse); 8) SRN (4 mg kg^−1^‐, based on CDK4/6i content) + T cells (1.5×10^7^ cells/mouse); 9) SRN‐T cells (1.5×10^7^ cells/mouse). Intravenous injection was given on days 7, 13 and 19 after tumor bearing, respectively. On day 21, the tumors and tumor extracts were collected. The tumor extracts were centrifugated and the supernatant was collected to detected the concentration of CCL5 and CXCL10 in tumor by ELISA kit following manufacturer's manual. The tumors were digested by collagenase IV (Sangon Biotech) to obtain a single‐cell suspension. Then, the lymphocyte was isolated by 40 to 70% Percoll (GE) gradient centrifugation and stained by FITC anti‐mouse CD3 antibody (1 µg/mL, Biolegend), APC/Cyanine7 anti‐mouse CD8a antibody (1 µg/mL, Biolegend), APC anti‐mouse CD45 antibody (1 µg/mL, Biolegend), and Brilliant Violet 421 anti‐mouse Thy1.1 antibody (1 µg/mL, Biolegend) to analyzed by flow cytometry to determine the quantitation of endogenous and exogenously transferred T cells in tumors.

In order to verify the mechanism of synergistic of SRN‐T cells in the tumor microenvironment, the tumor‐grafted mice were randomly divided into 9 groups with 4 mice in each group and given: 1) saline; 2) IDOi (3.5 mg kg^−1^); 3) CDK4/6i (4 mg kg^−1^); 4) IDOi (3.5 mg kg^−1^)+ CDK4/6i (4 mg kg^−1^); 5) SRN (4 mg kg^−1^, taking CDK4/6i content as reference); 6) T cells (1.5×10^7^ cells/mouse); 7) IDOi (3.5 mg kg^−1^) + CDK4/6i (4 mg kg^−1^) + T cells (1.5×10^7^ cells/mouse); 8) SRN (4 mg kg^−1^, based on CDK4/6i content)+ T cells (1.5×10^7^ cells/mouse); 9) SRN‐T cells (1.5×10^7^ cells/mouse). Intravenous injection was given on days 7, 13 and 19 after tumor bearing, respectively. On day 21, the tumors and tumor extracts were collected. The tumor extracts were centrifugated and the supernatant was collected to detected the concentration of Trp and Kyn in tumor by ELISA kit followed manufacturer's manual. The tumors were digested by collagenase IV (Sangon Biotech) to obtain a single‐cell suspension. Then, the lymphocyte and tumor cells were isolated by 40 to 70% Percoll (GE) gradient centrifugation.

Then, partly of the lymphocyte cells were stained by FITC anti‐mouse CD3 antibody (1 µg/mL, Biolegend), APC anti‐mouse CD4 antibody (1 µg/mL, Biolegend), Brilliant Violet 421 anti‐mouse Foxp3 antibody (1 µg/mL, Biolegend) and PE anti‐mouse Ki67 antibody (1 µg/mL, Biolegend) for 30 min, and washed twice with PBS to detected the infiltration and proliferation of regulatory T cells by flow cytometry.

Other lymphocyte cells were suspended the RPMI 1640 medium containing PMA (100 ng/mL), ionomycin (2 µM) and protein transport inhibitor BFA (10 µg/mL). After 4 h, the cells were collected, centrifuged at 300 g for 3 min to remove the supernatant and washed twice with pre‐cooled PBS. After staining with APC anti‐mouse CD3 antibody (1 µg/mL, Biolegend), Brilliant Violet 421 anti‐mouse Thy1.1 (0.5 µg/mL, Biolegend) and APC/Cyanine7 anti‐mouse CD8a (1 µg/mL, BD) antibody for 30 min, the cells were washed twice with pre‐cooled PBS. Then, the cells were fixed with 4% PFA at r.t for 10 min and perforated with 0.1% Triton for 10 min, and stained with PE/Cyanine 7 anti‐mouse GzmB antibody (8 µg/mL, Biolegend). Brilliant Violet 510 anti‐mouse IFNγ antibody (0.8 µg/mL, Biolegend) and PE anti‐mouse TNFα antibody (0.8 µg/mL, Biolegend) or 30 min. Flow cytometry was used to analyze the expression of cytokines of CD8^+^T cells and CD8^+^Thy1.1^+^ T cells including IFNγ, TNFα, and GzmB in the tumor microenvironment of mice after SRN‐T cells treated.

### Statistical Analysis

Statistical analyses were performed using GraphPad Prism 8.0. All plots show mean ± SEM. A one‐way analysis of variance (ANOVA) test with Tukey's correction was used for comparisons of multiple groups. A Student's unpaired *t* test was used for two‐group comparisons in the appropriate conditions. A log‐rank (Mantel‐Cox) test was used to analyze the statistical significance of difference for survival analysis. Statistical significance was set at ^*^ *p* < 0.05, ^**^ *p* < 0.01 and ^***^ *p* < 0.001, ^****^ *p* < 0.0001, and n.s denotes no significant difference.

## Conflict of Interest

The authors declare no conflict of interest.

## Supporting information

Supporting Information

## Data Availability

The data that support the findings of this study are available from the corresponding author upon reasonable request.
