# Supplementary material for: Spatiotemporally Controlled T‐Cell Combination Therapy for Solid Tumor
Source: Adv Sci (Weinh). 2024 Apr 17;11(25):2401100. doi: 10.1002/advs.202401100 (PMC11220647; doi:10.1002/advs.202401100)
Supplement: Supplementary file 1 — Supporting Information [file ADVS-11-2401100-s001.pdf]

ADVANCED SCIENCE
Open Access

Supporting Information

for *Adv. Sci.*, DOI 10.1002/advs.202401100

Spatiotemporally Controlled T-Cell Combination Therapy for Solid Tumor

Meixi Hao, Ying Zhou, Sijia Chen, Yu Jin, Xiuqi Li, Lingjing Xue, Mingxuan Shen, Weishuo Li and Can Zhang**

Supporting Information for

Spatiotemporally controlled T-cell combination therapy for solid tumor

Authors: Meixi Hao[‡], Ying Zhou[‡], Sijia Chen, Yu Jin, Xiuqi Li, Lingjing Xue, Mingxuan Shen, Weishuo Li,* and Can Zhang*

M. Hao, Y. Zhou, S. Chen, Y. Jin, X. Li, L. Xue, M. Shen, W Li, C. Zhang

State Key Laboratory of Natural Medicines, Center of Advanced Pharmaceuticals and Biomaterials, China Pharmaceutical University, Nanjing 211198, China. and Chongqing Innovation Institute of China Pharmaceutical University, Chongqing 401135, China.

E-mail: zhangcan@cpu.edu.cn

W. Li

Center for Molecular Metabolism, School of Environmental and Biological Engineering, Nanjing University of Science and Technology, 200 Xiao Ling Wei Street, Nanjing 210094, China.

E-mail: liweishuo@njust.edu.cn.

[‡] *These authors contributed equally to this work.*

Materials and Methods

Chemistry

To prepare GSH responsive liposomal core, lipidic CTA were designed and synthesized in this paper. Lipidic CTA is composed of hydrophobic tail chain double-stranded tetrahexanol, intermediate arm disulfide bond, and trithiol bond in the head. The hydrophobic tail chain can participate in the formation of the bimolecular skeleton of liposomes. The disulfide bond can respond to the high concentration of GSH in the cytoplasmic rupture to release drugs, and the trithiol bond can be used as a chain transfer agent to initiate the PET-RAFT polymerization reaction. These structures were confirmed by ^{1}H-NMR, MS or HRMS.

Synthesis of 2-aminoglutarate tetradecanol ester (**1**). Lutamic acid (2.9 g, 19.7 mmol) and p-toluenesulfonic acid (2.22 g, 11.7 mmol) were dissolved in 60 mL of toluene and refluxed for 1 h. Then, n-tetradecanol (5.0 g, 23.3 mmol) was added and refluxed for 12 h. Then, the solvent toluene was removed by vacuum distillation. Recrystallize the concentrated solution with 30 mL of methanol to obtain a white solid. This solid was dissolved in dichloromethane and washed with sodium bicarbonate aqueous solution (5%, 15 mL×2), and saturated salt water in sequence. Subsequently, the organic layer was separated, and dried over anhydrous Na_2SO_4 and evaporated. Then, 3.42 g Compound **1** was obtained as a white powdery solid (yield of 53.4%). MS, ESI$^+$, m/z: Calcd for $C_{33}H_{65}NO_4$ $(M+H)^+$, 539.49; Found, 540.50.

Synthesis of 1- (tert butoxycarbonyl) cysteamine (**2**). Cysteamine dihydrochloride (3.0 g, 13.3 mmol) was dissolved in 100 mL of methanol. Then, triethylamine (5.79 mL, 39.9 mmol) and a methanol solution of ditertbutyl ecarbonate ($(Boc)_2O$, 2.9 g, 13.3 mmol) were added in 0℃. The reaction mixture was stirred at r.t for 5 h, the reaction solvent was removed by vacuum distillation. The, 60 mL of 1M NaH_2PO_4 aqueous solution was added, and extracted with ether (50 mL×2).

The pH of the water layer was adjusted to 9.0 using a 1M NaOH aqueous solution, and then extracted with ethyl acetate (40 mL×2). The organic layer was separated, and dried over anhydrous Na_2SO_4 and evaporated. The residue was obtained to give Compound **2** as a white oily solid (1.57 g, 46.8%). MS, ESI^+, m/z: Calcd for $C_9H_{20}N_2O_2S_2$ $(M+H)^+$, 252.10; Found, 253.10.

Synthesis of 1- (tert butyloxycarbonyl) -8- (3-carboxylpropionyl) cysteamine (**3**). Compound **2** (1.57 g, 6.23 mmol) was dissolved in 60 mL of dichloromethane, then succinic anhydride (0.62 g, 6.23 mmol) and p-dimethylaminopyridine (DMAP, 0.23 g, 1.87 mmol) were added to the reaction solution, and then stirred at r.t for 5 h. After washed with 1M $KHSO_4$ aqueous solution (50 mL×2), the organic layer was collected and washed with saturated citric acid water and saturated salt water, and dried over anhydrous Na_2SO_4 and evaporated to give compound **3** as a white oily solid (2.12 g, 98.2%). ^1H NMR (500 MHz, $CDCl_3$) δ: 3.60 (m, 2H), 3.43 (s, 2H), 2.88 (m, 4H), 2.58 (m, 2H), 2.40 (m, 2H), 1.43 (s, 9H). MS, ESI^+, m/z: Calcd for $C_{13}H_{24}N_2O_4S_2$ $(M+Na)^+$, 352.11; Found, 375.10.

Synthesis of 2- (2,2-dimethyl-4,13-dioxy-3-oxa-8,9-disulfide-5,12-diazacetylamide) glutarate ditetradecanol diester (**4**). Compound **3** (2.12 g, 6.0 mmol), 1- (3-dimethylaminopropyl) -3-ethylcarbodiimide hydrochloride (EDCI, 2.31 g, 12.0 mmol) and 1-hydroxybenzotriazole (HOBt, 1.62 g, 12.0 mmol) were dissolved in 80 mL of chloroform and stirred at r.t for 3 h. Moreover, compound **1** (3.24 g, 6.0 mmol) and triethylamine (3.36 mL, 24.0 mmol) were dissolved in 40 mL of chloroform, stirred at r.t for 1 h, then the reaction mixture was added into the aforementioned mixed solution and stirred at r.t for 12 h. Then, the reaction mixture was washed with saturated salt water (80 mL×2) and the organic layer was dried over anhydrous Na_2SO_4. After evaporated the organic solution to obtain a white solid. The residue was purified by column chromatography (Dichloromethane: Methanol = 60: 1) to give Compound **4** as a white solid (4.1 g, 77.9%). ^1H

NMR (500 MHz, $CDCl_3$) δ: 4.57 (1H, m), 4.12 (2H, t, *J*=6.75), 4.06 (2H, t, *J*=6.80), 3.56 (2H, m), 3.43 (2H, m), 2.80 (4H, m), 2.57 (4H, m), 2.35 (2H, m), 2.19 (1H, m), 1.99 (1H, m), 1.62 (4H, m), 1.44 (9H, s), 1.26 (44H, m), 0.88 (6H, m). HRMS, ESI^+, m/z: Calcd for $C_{46}H_{87}N_3NaO_8S_2$ $(M+H)^+$, 873.5927; Found, 874.60017.

Synthesis of 2- (9-Oxo-4,5-Disulfide-1,8-Diazododecylamide) Bistetradecanol Glutarate Hydrochloride (**5**). Compound **4** (4.1 g, 4.7 mmol) was dissolved in 200 mL of saturated HCl dioxane solution, stirred at 0°C for 12 h. The reaction mixture was filtered and dried to obtain compound 5 as a white waxy solid (3.3 g, yield of 86.8%).

Synthesis of 2- ((Butylthioalkyl) - thiocarbonyl) - propionic acid (**6**). 2.7 g, 30 mmol of butanethiol was dissolved in 4.92 mL of 25% NaOH, then the solution was plated in a low-temperature reactor at 0°C. Subsequently, carbon disulfide (2.5 g, 33.8 mmol) was diluted with water and was slowly added into a NaOH solution of butyl mercaptan using syringe. The reaction mixture was stirred at 0°C for 30 min. Then, 2.7 mL of 2-bromopropionic acid (4.7 g, 30.8 mmol) was dissolved in 4.5 mL of 25% NaOH and was slowly added into the above reaction mixture. The reaction was stirred at r.t for 18 h and was plated at 0°C. Then, 30 mL of 10 M HCl solution was added and stirred for 30 min. After that, the reaction solution was washed with saturated salt water, dried over anhydrous Na_2SO_4 and evaporated. The yellow residue was purified by column chromatography (Dichloromethane: Methanol = 30: 1) to give Compound **6** as a yellow solid (2.1 g, 73.2%). ^{1}H NMR (500 MHz, CDCl3) δ: 8.69 (s, 1H), 4.86 (q, *J*=7.4 Hz, 1H), 3.37 (t, *J*=7.4 Hz, 2H), 1.74-1.65 (m, 2H), 1.62 (d, *J*=7.5 Hz, 3H), 1.45 (p, *J*=7.4 Hz, 2H), 0.94 (t, *J*=7.4 Hz, 3H). MS, ESI^+, m/z: Calcd for $C_8H_{14}O_2S_3$ $(M+H)^+$, 238.02; Found, 239.02.

Synthesis of N_2-(tert butoxycarbonyl)-N_6-(2 (((butyl) carbonyl) thio) propionyl) lysine (**7**). Compound **6** (1.56 g, 6.6 mmol), EDCI (2.01 g, 10.5 mmol), and N-hydroxysuccinimide (NHS,

1.21 g, 10.5 mmol) were dissolved in 80 mL of chloroform and stirred at r.t for 3 h. Meanwhile, *N*-tert-butyloxycarbonyl lysine (Boc-Lys-OH, 1.61 g, 6.6 mmol) and *N*, *N*-di-isopropylethylamine (DIPEA, 3.3 mL, 26.2 mmol) were dissolved in 40 mL of chloroform, stirred at r.t for 1 h and were added into the above reaction mixture. Then, the reaction mixture was stirred at r.t for 12 h, and was washed with saturated salt water. After dried over anhydrous Na_2SO_4, the organic layer was evaporated and to obtain a yellow solid. Then, the yellow crude was purified by column chromatography (Dichloromethane: Methanol=30: 1) to obtain Compound **7** as a white solid (3.4 g, 72.6%). ^{1}H NMR (500 MHz, $CDCl_3$) δ: 4.71 (q, *J*=7.3 Hz, 1H), 4.25 (s, 1H), 3.46-3.30 (m, 2H), 3.30-3.14 (m, *J*=6.8 Hz, 2H), 1.83 (s, 1H), 1.72-1.64 (m, 3H), 1.57 (s, 3H), 1.50 (d, *J*=7.0 Hz, 2H), 1.44 (s, 9H), 1.41 (d, *J*=7.3 Hz, 3H), 1.39-1.34 (m, 2H), 0.93 (t, *J*=7.3 Hz, 3H). MS. ESI$^+$, m/z: Calcd for $C_{19}H_{34}N_2O_5S_3$ $(M+Na)^+$, 466.16; Found, 489.15.

Synthesis of diethyl (15- ((tert butoxycarbonyl) amino) -8-methyl-9,16,25-trioxy-6-thioxy-5,7,20,21-tetrathio-10,17,24-triazaoctadecane-28-acyl) glutamate (TA_2-Glu-Suc-AED-Boc-Lys-OH-S_3, **CTA lipidic, 8**). Compound **7** (1.3 g, 2.8 mmol), EDCI (0.85 g, 4.5 mmol), and NHS (0.51 g, 4.5 mmol) was dissolved in 80 mL of chloroform and stirred at r.t for 3 h. Meanwhile, compound **5** (2.2 g, 2.8 mmol) and DIPEA (1.8 mL, 13.9 mmol) was dissolved in 40 mL of chloroform and stirred at r.t for 1 h and were added into the above reaction mixture. Then, the reaction mixture was stirred at r.t for 12 h, and was washed with saturated salt water. After dried over anhydrous Na_2SO_4, the organic layer was evaporated and to obtain a yellow solid. Then, the yellow crude was purified by column chromatography (Dichloromethane: Methanol=25: 1) to obtain Compound **8** as a white solid (2.4 g, 76.2%). ^{1}H NMR (500 MHz, $CDCl_3$) δ: 4.76 (m, 1H), 4.13 (dd, *J*=14.1, 6.7 Hz, 2H), 4.06 (t, *J*=6.9 Hz, 1H), 3.69-3.51 (m, 2H), 3.38 (q, *J*=7.5 Hz, 2H), 3.23 (s, 2H), 2.90-2.74 (m, 3H), 2.57 (s, 2H), 1.72-1.55 (m, 17H), 1.51 (s, 2H), 1.45-1.41 (m, 12H), 1.26 (s, 49H), 0.94 (t,

J=7.4 Hz, 3H), 0.88 (t, *J*=6.8 Hz, 6H). MS, ESI^+, m/z: Calcd for $C_{60}H_{11}1N_5O_{10}S_5$ $(M+H)^+$, 1221.69; Found, 1222.70.

Isolation of mouse $CD8^+$T cells

Spleens from C57BL/6J Thy1.1$^+$ pmel-1 mice were ground through a 70-μm filter, and red blood cells were removed by incubation with ACK lysis buffer for 5 min at 4°C. Then, the splenic cells were centrifuged, washed with D-PBS (without Ca^{2+} and Mg^{2+}), and isolated by EasySep^{TM} Mouse $CD8^+$T cell Isolation Kit (StemCell) to obtain naïve pmel-1 Thy1.1$^+$ $CD8^+$T cells ($CD8^+$T cells). For activated $CD8^+$T, naive $CD8^+$T cells were resuspended at 1.5×10^6 cells per milliliter in RPMI medium containing 10 ng/mL recombinant mouse IL-2, plate-bound 5 μg/mL anti-CD3, and 2 μg/mL anti-CD28 agonist antibodies and incubated at 37°C. After an incubation period of 2 days, dead cells were removed by centrifugation and living cells were collected. Activated $CD8^+$T cells were cultured in a medium containing IL-2 and anti-CD3 and anti-CD28 agonist antibodies for *in vitro* and *in vivo* studies.

Preparation and characterization of SRN

Liposomal core was prepared by using a film dispersion method. Briefly, 50 mg natural soybean phosphatidylcholine (S100), 9 mg cholesterol (Chol), 3.5 mg CDK4/6i and 12 mg lipidic CTA were dissolved in 5 mL of mixture of $CHCl_3$ and MeOH (3: 2, *v: v*). After the organic solvents were evaporated at 40°C, a thin lipid film was formed. Then the organic solvents were then further removed under vacuum overnight. The lipid film was hydrated with 3.5 mL of the distilled water at 37°C for 30 min, then the liposomes were obtained by dispersion using an ultrasonic cell disruptor under ice bath (30% power, 15 min) and extrusion through polycarbonate membrane filters with a pore size of 0.45 μm.

Using the PET-RAFT polymerization reaction to constructe SRN. Berifly, PET-RAFT polymerization was carried out in pH 7.4 aqueous solution to build an acid responsive gel layer containing IDOi on the surface of liposomal core as the shell of SRN. Berifly, acrylamide (4 mg, 0.056 mmol), glycerol methacrylate (1.23 mg, 5.396 μmol), and DBCO-PEGA (386 μg, 386 μ mol) were added into 650 μL aqueous solution of liposomal core. The mixture was placed in a 2 mL transparent glass container with a rubber diaphragm. Then, 1% triethanolamine aqueous solution (10.4 μL, *v: v*), 0.5 mg/mL Eosin Y aqueous solution (15.0 μL) and 1 mg of IDOi were added to the glass container, repectively. Then, the reaction system was thoroughly stirred and mixed. Then, a 27 G, 1/2 needle was connected to a nitrogen (N_2) source and was inserted through a rubber spacer to blow N_2. Moreover, another 27 G, 1/2 needle was inserted through the spacer to vent for 10 min. Then, the reaction mixture was stirred in a dark at 250 rpm for 10 min, and turn on the 465 nm (10 mW/cm^2) light source, which act the polymerize reaction for 30 min and obtain SRN with a shell core structure. The liposomal core and SRN were diluted to a certain concentration to observe the morphology and particle size by TEM and Dynamic light scattering (DLS) analyzer (Brookhaven).

To investigate the stepwise degraded of SRN, SRN was incubated with PBS (pH 7.4), PBS (pH 6.5), and PBS (pH 6.5) containing 10 mM GSH. After incubated at 37°C for 4 h, SRN solutions were collected and placed in a dialysis bag with molecular weight cut off 8000-12000 Da. Then, the morphology of nanoparticles in each group was observed and photographed by TEM.

To investigate the stability of SRN, particle size was monitored of SRN after incubated with 1640 medium or 10% FBS in 1640 medium for different periods (0, 0.5, 1, 2, 4, 6, 10, 24, h). Meanwhile, drug loading stability and particle size changes of SRN under different temperature was also evaluated for different time (24, 48, 72 h) using HPLC and DLS.

To investigate the IDOi and CDK4/6i release from SRN under physiological conditions *in vitro*，normal blood environment (PBS, containing 0.01 mM GSH) were applied as a dialysis medium. Putting a dialysis bag (molecular weight cut-off of 8000-12000 DA) containing 1 mL SRN of 500 μg/mL CDK4/6i into 50 mL solution of the above physiological conditions, respectively, and incubated at 37℃ in a water bath thermostatic shaker. The sample in of each dialysis bag was collected at different time (0, 0.5, 1, 2, 4, 6, 8, 12, 24, 48 h) for HPLC detection after centrifuging to supernatant, and the cumulative release amounts of CDK4/6i and IDOi at different time points were calculated and the release profiles were plotted.

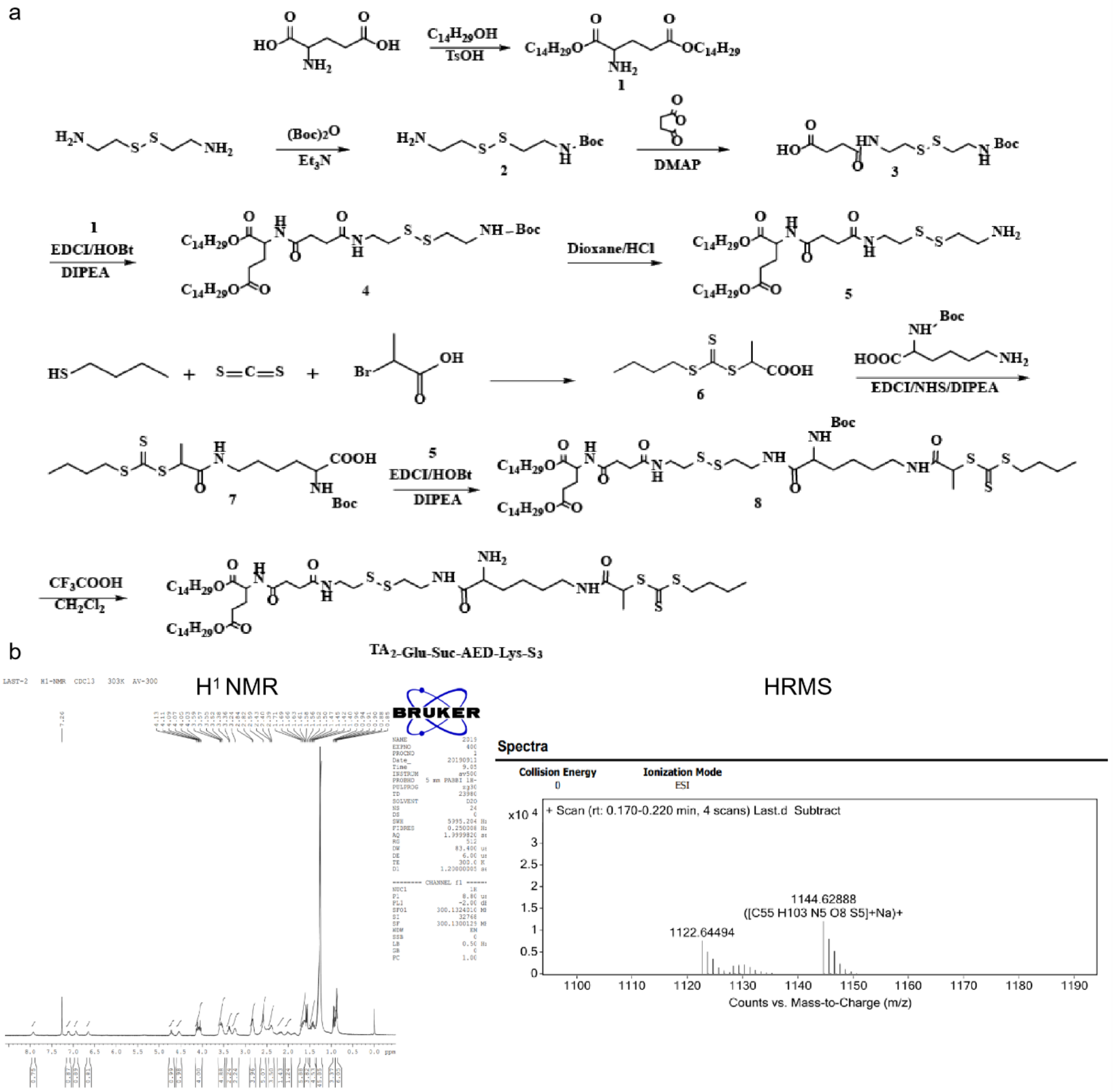

Figure S1. The synthetic and the ^{1}H-NMR and HRMS of lipidic CTA (TA_2-Glu-Suc-AED-Lys-S_3).

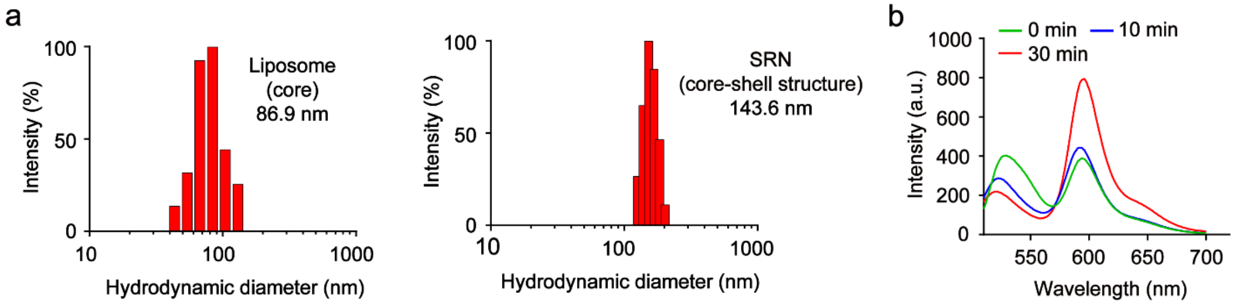

Figure S2. Characterization of SRN structures. a. The hydrodynamic sizes of liposomal core and SRN. **b.** The colorimetric intensity of the colored hydrogels at different wavelengths *via* the treatment of photoinitiator Eosin Y and photocatalytic conditions.

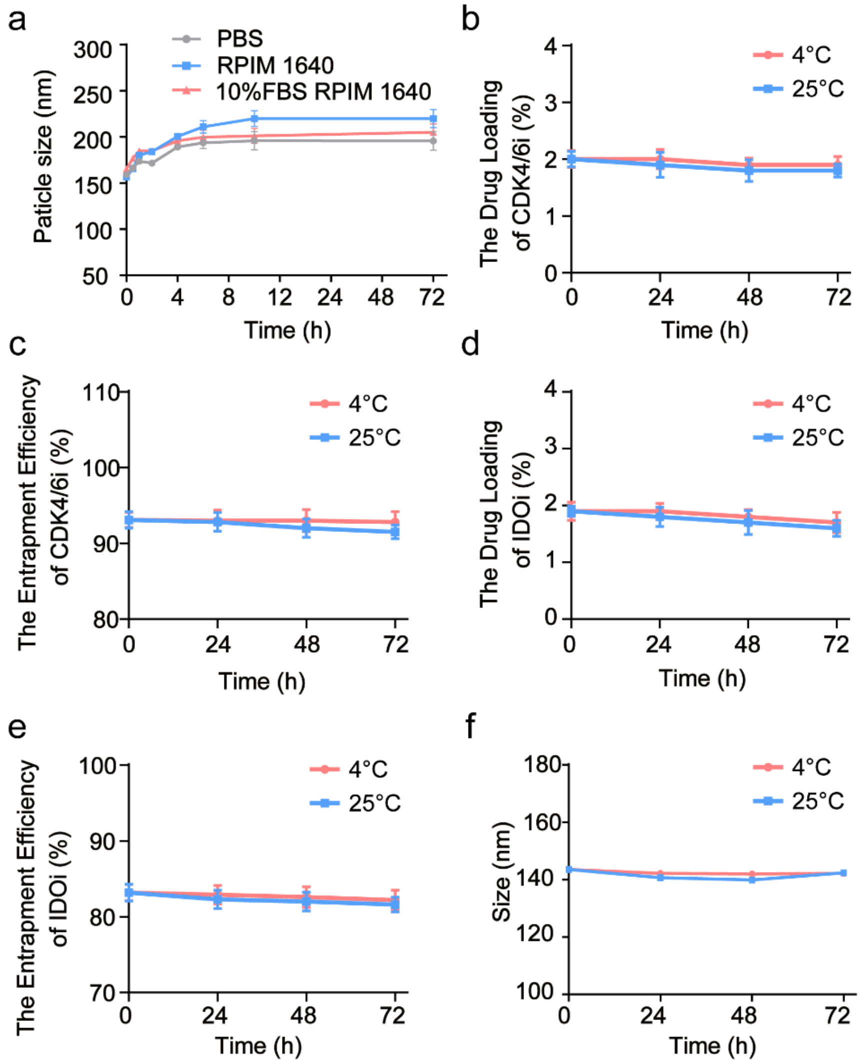

Figure S3. The stability of SRN at different conditions. a. Particle size changes of SRN in different physiological environments. **b-e.** Drug loading stability of SRN under different temperature. **f.** Particle size changes of SRN under different temperature. Data were shown as mean ± SEM (n = 3).

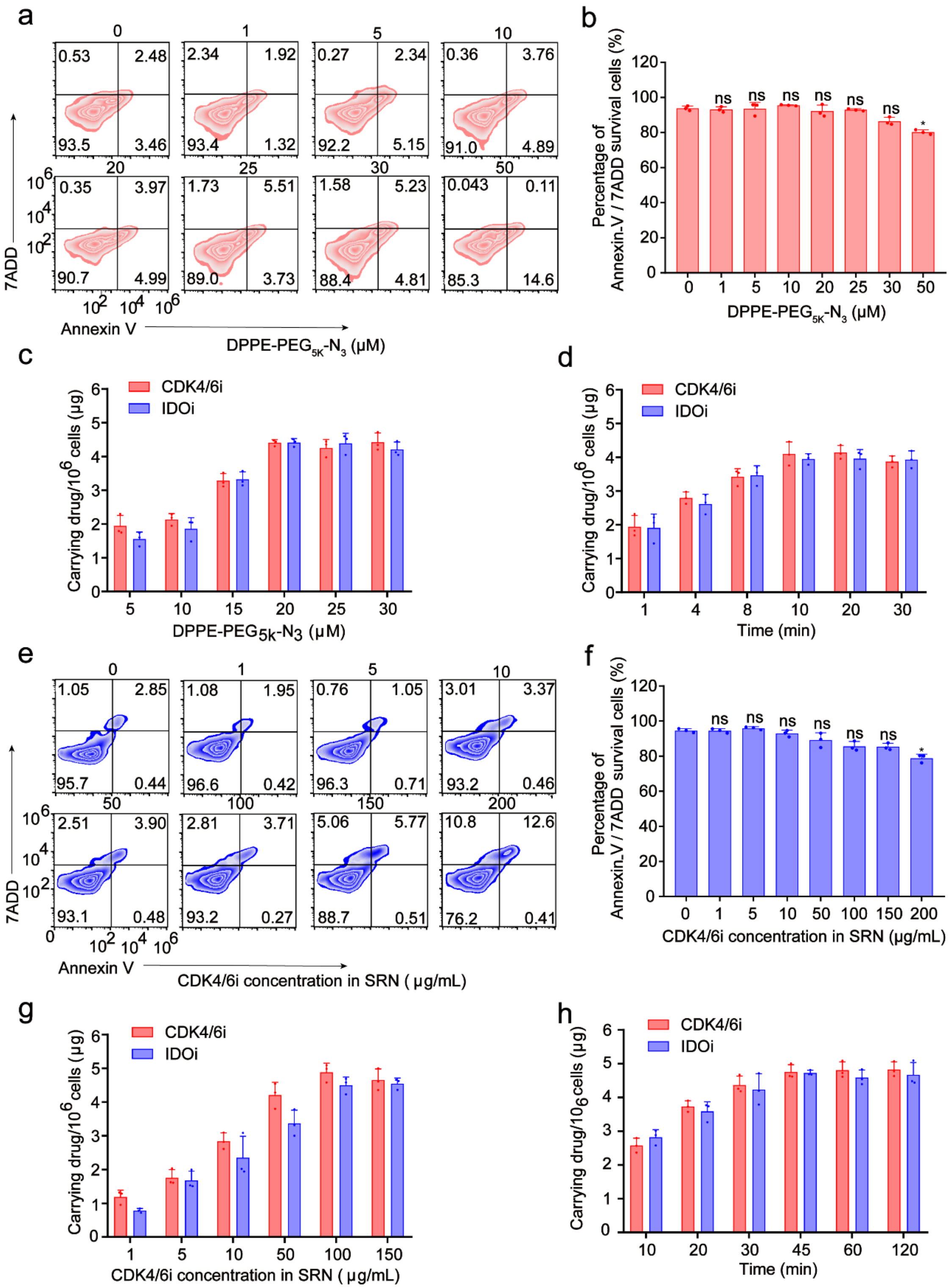

Figure S4. Optimize the conditions for preparation of SRN-T cells. a, b. *In vitro* cytotoxicity of different concentration of DPPE-PEG$_{5k}$-N$_3$ on CD8$^+$T cells for 24 h. **c.** The cells were incubated

with DPPE-PEG$_{5k}$-N$_3$ for different concentration. **d.** The cells were incubated with 20 μM DPPE-PEG$_{5k}$-N$_3$ for different time. **e, f.** *In vitro* cytotoxicity of different concentration of SRN on CD8$^+$T cells for 24 h. **g.** The cells were incubated with SRN for different concentration. **h.** The cells were incubated with SRN for different time. Data are represented as mean ± SEM; n = 3; **P* <0.05; n.s, not significant; determined by one-way ANOVA with Tukey's correction in **b**, **f**.

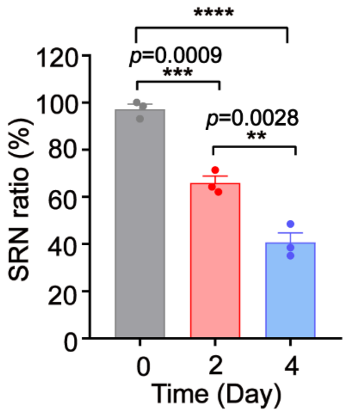

Figure S5. Stability of SRN-T cells in context of SRN anchorage. Data are represented as mean ± SEM; n = 3; ***P* < 0.01; ****P* < 0.001; *****P* < 0.0001; determined by one-way ANOVA with Tukey's correction.

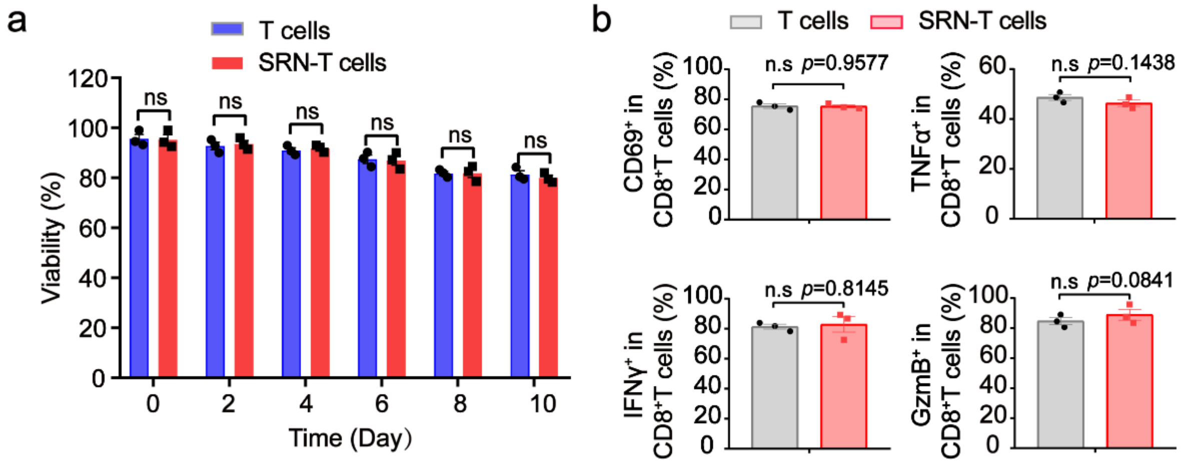

Figure S6. The cell viability and activation level of SRN-T cells. a. Histogram of the cell viability of T cells during the CTLs expanded. **b.** Histogram of the activation level of SRN-T cells. Data are represented as mean ± SEM; n = 3; n.s, not significant; determined by one-way ANOVA with Tukey's correction in **a** or unpaired *t* test in **b**.

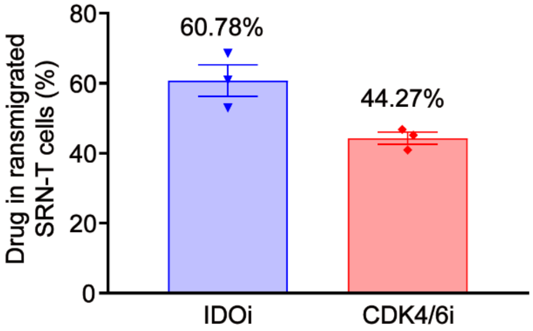

Figure S7. Quantification of CDK4/6i and IDOi on the SRN-T cells after transmigrated HUVEC cell monolayers in the presence of MCP-1 (20 ng/mL) for 12 h. Data were shown as mean ± SEM (n =3)**.**

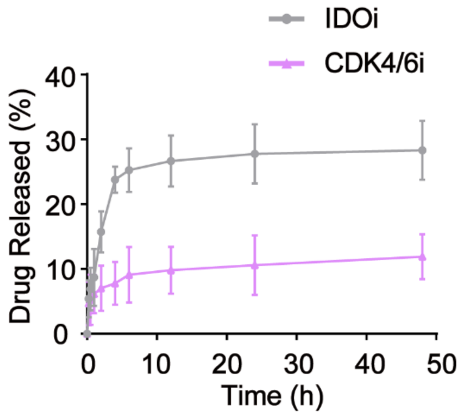

Figure S8. Stability of IDOi and CDK4/6i remained on SRN-T cells under physiological conditions. Data were shown as mean ± SEM (n = 3).

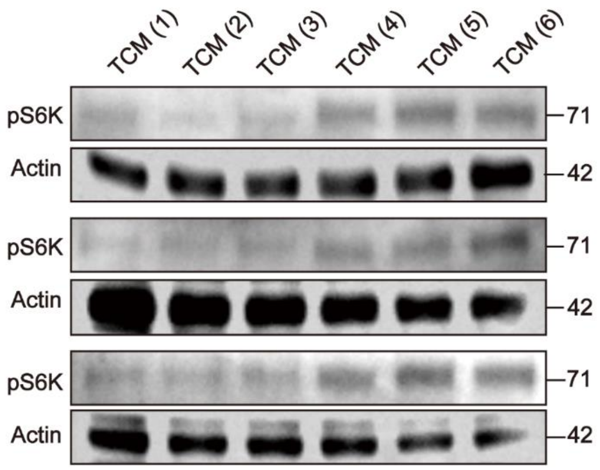

Figure S9. Triple independent experiments of Western blot.

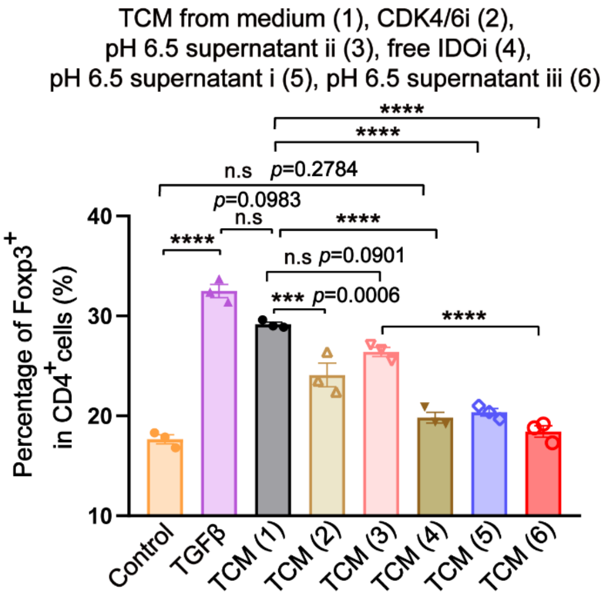

Figure S10. Quantification of Treg in CD4^{+}T cells. Data are represented as mean ± SEM; n = 3; ****P* < 0.001; *****P* < 0.0001; n.s, not significant; determined by one-way ANOVA with Tukey's correction.

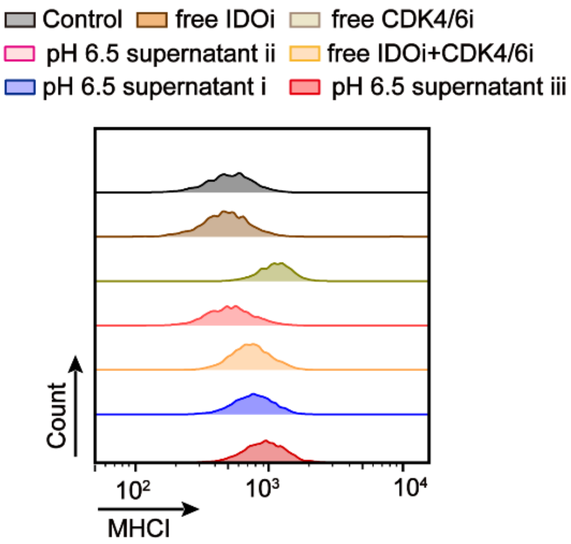

Figure S11. Representative flow cytometric analysis of the level of MHC-1 expression of B16F10 cells.

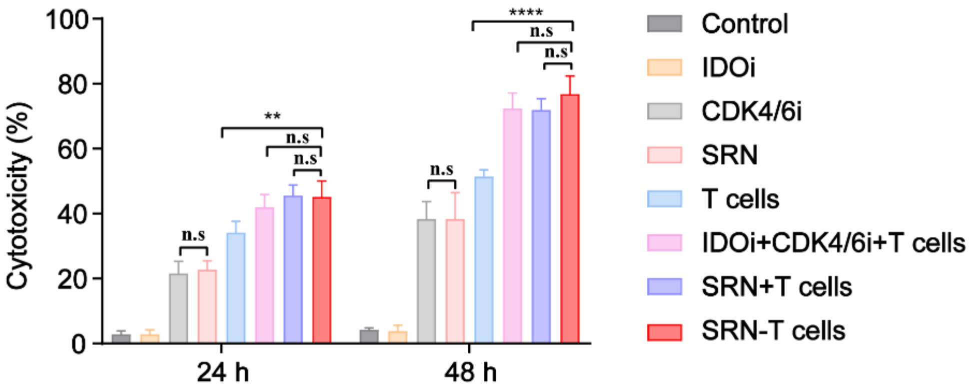

Figure S12. The cytotoxicity of free IDOi, free CDK4/6i, SRN, T cells, T cells plus free drugs, T cells plus SRN, SRN-T cells on B16F10 cells. Data are represented as mean ± SEM; n = 3; $**P < 0.01$; $****P < 0.0001$; n.s, not significant; determined by one-way ANOVA with Tukey's correction.

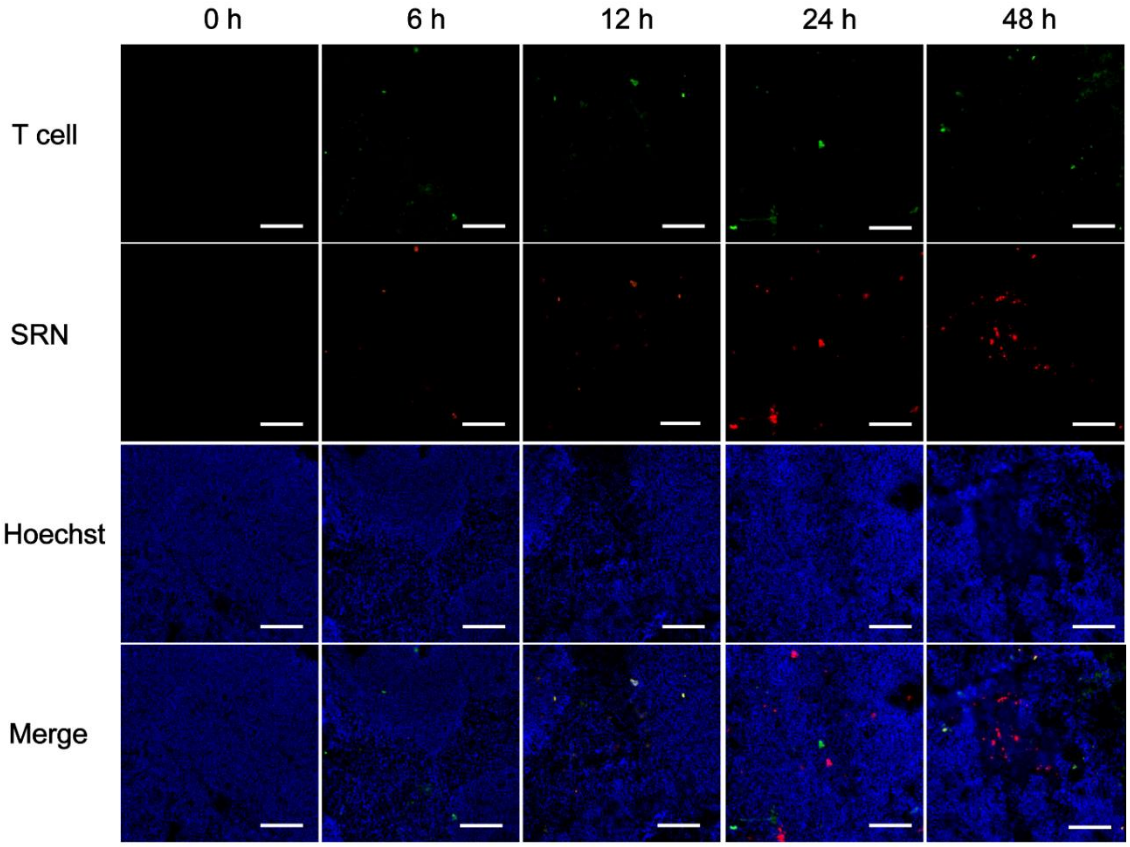

Figure S13. The tumor sections after administrated SRN-T cells for different time. T cells were labelled with CFSE and SRN were labelled with Rhodamine B. Scale bar, 200 μm.

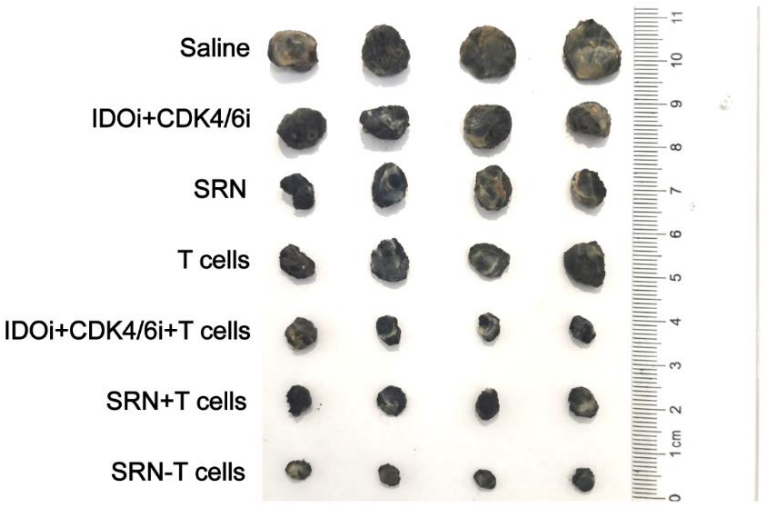

Figure S14. The digital image of tumors after resection (n = 4).

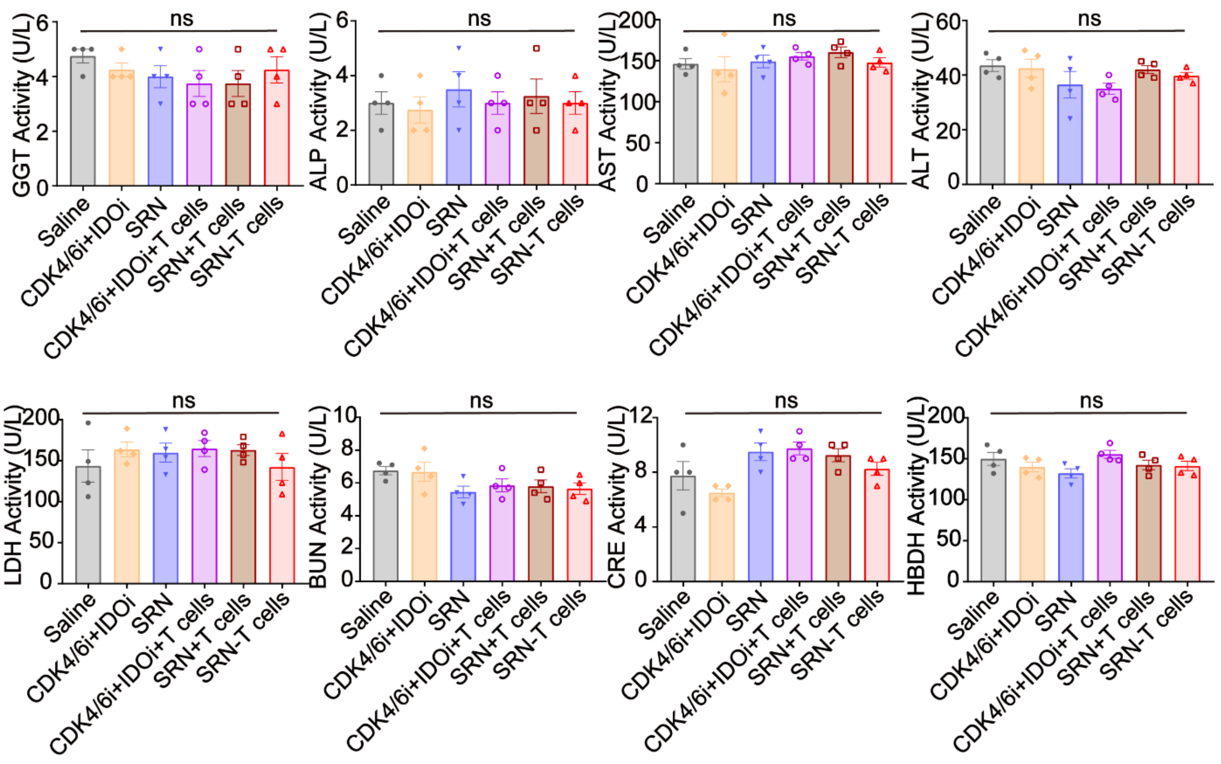

Figure S15. Biosafety analysis of different treatment groups in B16F10 tumor-bearing mice. n = 4 mice/group. Data are represented as mean ± SEM; n.s, not significant; determined by one-way ANOVA with Tukey's correction.

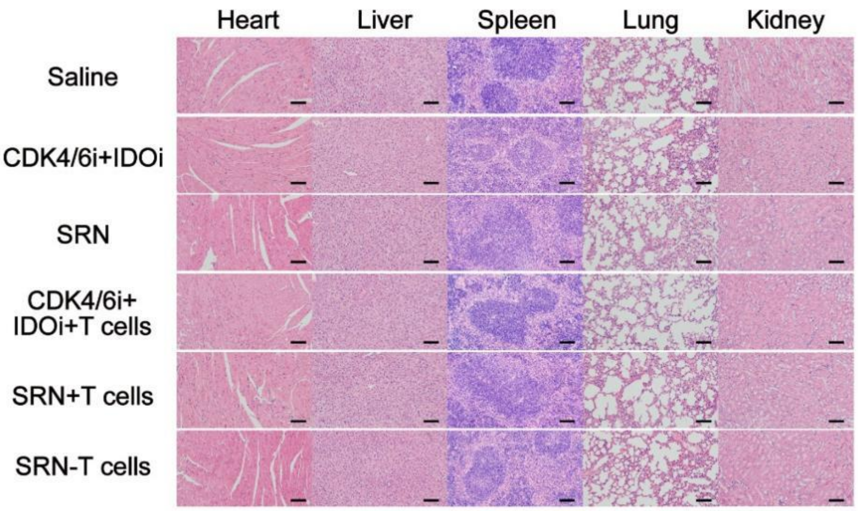

Figure S16. The Safety evaluation of heart, liver, spleen, lung, and kidney tissue by microscopic pathological analysis in B16F10 orthotopic melanoma tumor-bearing mice. Tissues were collected from B16F10 tumor-bearing mice after administration of different treatments. Hematoxylin and eosin (H&E) staining was performed on tissue sections for pathological assessment. Scale bar, 200 μm.

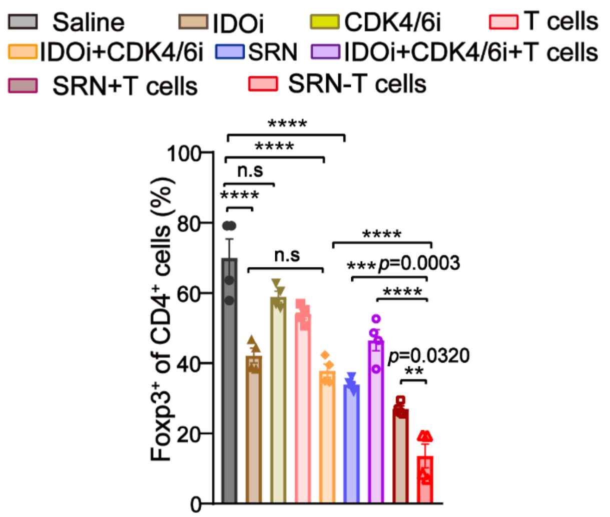

Figure S17. Histogram of the amount of Treg in CD4$^+$T cells. Data are represented as mean ± SEM; n = 4 mice/group; ***P* < 0.01; ****P* < 0.001; *****P* < 0.0001; n.s, not significant; determined by one-way ANOVA with Tukey's correction.
